# From Dormancy to Eradication: Strategies for Controlling Bacterial Persisters in Food Settings

**DOI:** 10.3390/foods14061075

**Published:** 2025-03-20

**Authors:** Susana Serrano, Mirjana Ž. Grujović, Katarina G. Marković, Maria Teresa Barreto-Crespo, Teresa Semedo-Lemsaddek

**Affiliations:** 1 CIISA—Centre for Interdisciplinary Research in Animal Health, Faculty of Veterinary Medicine, University of Lisbon, 1300-477 Lisbon, Portugal; sserrano@fmv.ulisboa.pt; 2 Associate Laboratory for Animal and Veterinary Sciences (AL4AnimalS), 500-801 Vila Real, Portugal; 3 Department of Science, Institute for Information Technologies Kragujevac, University of Kragujevac, Jovana Cvijića bb, 34000 Kragujevac, Serbia; katarinam@kg.ac.rs; 4 iBET, Institute of Experimental Biology and Technology, 2781-901 Oeiras, Portugal; tcrespo@ibet.pt; 5 ITQB, Institute of Chemical and Biological Technology António Xavier, Nova University of Lisbon, Republic Avenue, 2780-157 Oeiras, Portugal; 6 BioISI—Biosystems & Integrative Sciences Institute, Faculty of Sciences, University of Lisbon, 1749-016 Lisbon, Portugal

**Keywords:** persister cells, environmental triggers, food industry, eradication strategies

## Abstract

Bacterial persistence, a dormant state that enables microorganisms to survive harsh conditions, is a significant concern in food-industry settings, where traditional antimicrobial treatments often fail to eliminate these resilient cells. This article goes beyond conventional review by compiling critical information aimed at providing practical solutions to combat bacterial persisters in food production environments. This review explores the primary mechanisms behind persister cell formation, including toxin–antitoxin systems, the alarmone guanosine tetraphosphate (ppGpp), stochastic processes (in which persistence occurs as a random event), and the SOS response. Given the serious implications for food safety and quality, the authors also report a range of physical, chemical, and biological methods for targeting and eradicating persister cells. The strategies discussed, whether applied individually or in combination, offer varying levels of availability and applicability within the industry and can serve as a guide for implementing microbial contamination control plans. While significant progress has been achieved, further research is crucial to fully understand the complex mechanisms underlying bacterial persistence in food and to develop effective and targeted strategies for its eradication in food-industry settings. Overall, the translation of these insights into practical applications aims to support the food industry in overcoming this persistent challenge, ensuring safer, more sustainable food production.

## 1. Introduction

To survive and proliferate, every living organism must develop strategies to cope with environmental and cellular changes. Failure to do so can lead to the extinction of a species. Microorganisms are no exception. Apart from other mechanisms of defense to withstand environmental stresses, microorganisms can enter dormancy states to survive under adverse conditions. These states include bacterial cell persisters, viable but non-culturable (VBNC) cells, and resistant cells, each with distinct characteristics that contribute to their survival and adaptation [1,2,3,4].

Persister cells were first described by Joseph Bigger in 1944 during resistance assays using penicillin against *Staphylococcus* spp. [5]. The author observed a small subset of cells that survived despite not being resistant to penicillin. These survivor cells, though few in number, regained metabolic activity after the removal of the antibiotic. Bigger proposed that these cells entered a dormant state, preventing them from engaging in cellular pathways targeted by penicillin and allowing them to remain viable under stress conditions that would typically kill actively growing cells.

Later, in 1983 and 1986, Moyed and Bertrand focused on identifying the genes responsible for persistence, using *Escherichia coli* as a model [6,7]. These authors identified two distinct colony types when exposed to ampicillin: resistant cells and highly persistent mutant (Hip) persister cells. Unlike resistant cells, Hip persister cells did not grow in the presence of ampicillin. This discovery suggested a new phenotype of cells emerging in the stationary phase that was not linked to genetic mutations, revealing a unique survival strategy.

The studies mentioned above suggest that dormancy can be triggered by various factors, such as environmental stress (e.g., starvation, extreme pH, temperature, or salinity), antimicrobial pressure, and internal stressors like oxidative stress and DNA damage. However, alternative theories propose that persister cells may emerge from population heterogeneity, with stochastic errors during replication contributing to their formation [8,9,10,11]. Initial exposure to antibiotics causes rapid death in most cells, but a small subset of persisters survive and eventually resume growth once the stressor is removed. This persistence is often reflected in a “tail” effect, characterized by a slower, power-law decline in population size during stress, followed by regrowth once conditions improve [11].

Another study suggested that bacterial populations consist of both persister and non-persister cells [10]; non-persisters contribute to environmental detoxification, allowing persisters to survive and repopulate. The time required for detoxification is determined by the density of detoxifying cells, leading to a slower decline and the eventual recovery of persisters.

The comprehension of the genetic basis of persister has been recently studied by Blattman et al. Studies with *E. coli* revealed key physiologic and genetic factors that underline starvation-triggered persistence, a critical step towards targeting persisters in recalcitrant bacterial infections [12].

In food settings, the presence of persister cells poses a challenge for food safety and public health. These cells can survive food processing methods, as well as disinfection and sanitation procedures that typically eliminate active bacterial populations, leading to potential foodborne illnesses if pathogens are present. Therefore, this review begins by addressing the most relevant hypotheses for persister cell formation, including mechanisms like toxin–antitoxin systems, the alarmone guanosine tetraphosphate (ppGpp), stochastic processes, and the SOS response. It follows up with a detailed description of various physical, chemical, and biological approaches to target and eliminate persister cells. Considering the significant impact of persistence on food safety and quality, this manuscript aims to relate food-industry settings with persisters formation to better understand how to apply the different eradication techniques. The strategies presented, whether used independently or in combination, exhibit different levels of feasibility and relevance for the industry and may provide valuable guidance for implementing microbial contamination control measures.

## 2. Literature Search Strategy

To ensure a comprehensive and up-to-date review of bacterial persistence in food settings, we conducted a structured literature search using PubMed, Scopus, Web of Science, and Google Scholar for a broader search, followed by a directed one using connected papers, as well as ResearchRabbit. The search covered studies published between 2000 and 2024, focusing on peer-reviewed journal articles, reviews, and relevant industry reports. The keywords and search terms included “bacterial persisters”, “persistent cells”, “food industry”, “foodborne pathogens”, “eradication strategies”, “biofilms”, “antimicrobial resistance”, “persisters formation”, and combinations thereof. Boolean operators (and, or) were used to refine the search and ensure the inclusion of relevant publications. Additional sources were identified through the cross-referencing of key articles. This approach allowed us to compile a balanced and representative selection of studies addressing the mechanisms, challenges, and potential eradication strategies for bacterial persisters in food production environments.

## 3. Formation, Survival, and Regrowth

Persistence, as mentioned, is a physiological state in which small subpopulations of bacteria transiently assume a pronounced non-heritable stress-resilient phenotype. Although a universal definition is lacking, persisters are often described as transiently antibiotic-tolerant phenotypic variants that allow a population to survive antibiotic exposure. Although the physiological mechanisms conferring that increasing fitness can be very complex, one of the main features of persisters is a low metabolic state that may arise due to environmental triggers [13], which does not mean that persisters are completely metabolically inactive. Moreover, persister cells have been identified in almost every bacterial species examined, at levels covering several orders of magnitude, typically between 0.001% and 1% [13]. These have been identified in both Gram-positive and Gram-negative bacteria, as well as in eukaryotes such as yeast, where they exhibit tolerance to antifungal agents [13]. Persistence represents a formidable obstacle to antibiotic treatment since these molecules normally require strongly metabolically active cells to exert their antibacterial effects [14].

However, there is no scientific consensus on the mechanisms driving the formation of persister cells. To date, persisters are broadly classified into three types: type I, type II and type III based on their formation mechanisms. Type I persisters appear in the stationary phase in response to environmental triggers [1,2,3,4,5,6,7], while type II persisters are stochastically generated throughout the exponential phase [8,9,10,11,14,15]. A potential third class of persisters, type III persisters, referred to by Urbaniec et al. [16] as “specialized persisters,” has also been observed. Type III persisters are not slow-growing prior to antibiotic exposure and often exhibit persistence mechanisms specific to particular antibiotics, as described by Wakamoto et al. [17], as well as Goormathigh and Van Melderen et al. [18]. Therefore, understanding these mechanisms is essential for devising effective strategies to eliminate persisters and enhance food safety.

## 4. Types of Persisters

### 4.1. Type I or Triggered Persisters

Type I persisters are a subpopulation of bacterial cells that emerge during the stationary phase, typically triggered by environmental stress. These cells enter a reversible, dormant state that allows them to survive under harsh conditions, including exposure to antimicrobials [19]. The study of this type of persisters was conducted by Balaban et al. [15] using *E. coli*; the authors linked *E. coli* persistence to inherent population heterogeneity. For type I persisters, the authors investigated *hipA7* mutants and observed that, following antibiotic exposure and subsequent removal, persister cells initially in growth arrest could switch to actively dividing cells and repopulate the environment with antibiotic-sensitive microorganisms. Consequently, the authors defined type I persisters in *E. coli* as a preexisting subpopulation of non-growing cells generated during the stationary phase. Type I persisters may also be explained through bet-hedging theory, which posits that maintaining different phenotypes within a population increases survival chances when unfavorable conditions arise [20].

### 4.2. Type II or Stochastic Persisters

Type II persisters in *E. coli* were also studied by Balaban et al. [15]. The authors analyzed *hipQ* mutants and found that *hipQ* persisters differ from type I persisters. Unlike type I persisters, type II persisters do not undergo growth arrest. Instead, they continuously grow within the population, albeit at a significantly slower rate than non-persisters.

### 4.3. Type III or Specialized Persisters

Specialized persisters do not rely on slow growth or reduced metabolic rates to survive antibiotic exposure. Instead, they exhibit persistence mechanisms specific to particular antibiotics. These cells can arise spontaneously, as observed in *Mycobacteria*, where stochastically low levels of the enzyme catalase–peroxidase—responsible for activating isoniazid—enable persistence [17]. Alternatively, they can be induced via stress signals, such as ciprofloxacin persisters, which are triggered when *E. coli* is exposed to this antibiotic [18,20].

## 5. Mechanisms of Persister Cell Formation

There are several molecular mechanisms involved in the formation of persister cells and their tolerance to antibiotics. These mechanisms include the toxin–antitoxin (TA) system, the (p)ppGpp network, the quorum sensing (QS) system, drug efflux pumps, reactive oxygen species (ROSs), the SOS response, and RpoS (a sigma factor associated with the stationary phase), among others. Each of these systems plays a crucial role in modulating bacterial survival under stressful conditions, enabling the population to persist despite antibiotic exposure [21].

### 5.1. Toxin–Antitoxin System-Induced Persisters

The mediation of persistence has been proposed as a function of toxin–antitoxin (TA) systems [22]. TA systems are widespread across bacteria and archaea, defined as ubiquitous small operons containing two genes that separately express a stable toxin molecule to slow down/block certain metabolic processes and a labile antitoxin to neutralize this toxicity, which can be RNA or a protein [23,24]. There are eight groups (types I to VIII) of TA systems. Except for type VIII, in which the toxins are RNAs, in all other TA classes, the toxins are proteins. Antitoxins are either non-coding RNAs (types I, III, and VIII) or proteins (types II, IV, V, VI, and VII) [25,26]. Persisters are mostly associated with type I and type II TA systems.

**Figure 1:**
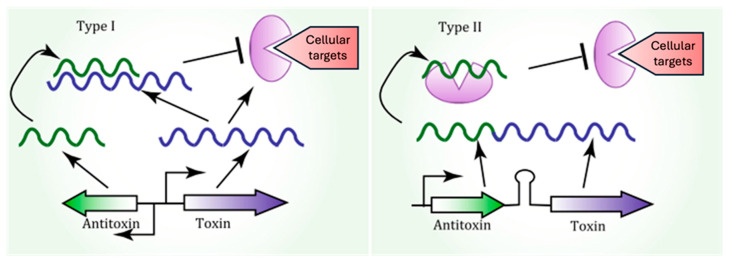
Schematic representation of the modes of action type I and type II TA systems. Figure adaptation from Zhang et al. [27] review.

Zhang et al. [27], in their review, consider that type II is probably the most abundant, as well as the best characterized class of TAs. These contain many families of toxins with different molecular activities such as kinases, ribonucleases (ribosome and ribosome-independent), acetyltransferases, and gyrase inhibitors. Type I TA systems are made of a small regulatory RNA, as an antitoxin, and a mRNA as an antitoxin (Figure 1) [28]. The better-characterized type I TA systems are Hok/Sok, TisB/IstR, and LdrD/RdID, SymE/SymR, which are usually involved in the disruption of the cell wall and transcription and translation, respectively [28]. Type II TA systems, known for their redundancy, have been linked to persister cell formation through protein–protein interactions. The HipA–HipB complex was the first TA system associated with persistence, specifically in *E. coli*, where the overexpression of the *hipA* gene increases persistence, while its deletion reduces it [4]. Other TA systems, including mqsRA and yafQ, have also been implicated in the reduction in *E. coli* survival during antibiotic treatment. Recent reviews by Zhang et al. [27] have linked other TA complexes, such as RelE, MazF, YafO, and VapC, to persistence. Other knockout library studies have identified additional candidate genes associated with persisters, including global regulators like DskA, DnaKJ, or IhfAB; this topic has been reviewed by Pizzolato-Cezar et al. [25].

### 5.2. Stringent Response

**Figure 2:**
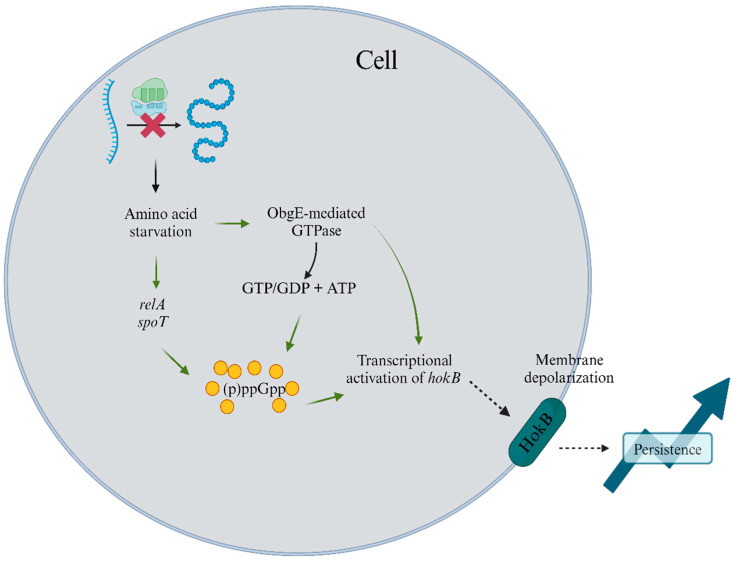
Production pathways of ppGpp and its activation of the type I TA system of HokB–SokB. An amino acid starvation triggers a stringent response, leading to (p)ppGpp accumulation and the subsequent activation of the HokB–SokB type I TA system. The HokB toxin disrupts membrane potential, contributing to bacterial persistence and survival under stress conditions. The full arrows indicate that it is direct effect. The dotted ones indicate a more indirect effect. In the case of membrane depolarization, the HokB membrane-associated toxin must be activated by the anti-toxin SokB in order to create the pores on the membrane creating the depolarization, which in turn will trigger the increase in persistence.

The stringent bacterial response is orchestrated via the stress alarmone ppGpp. This alarmone acts as a secondary messenger, modifying gene transcription in response to environmental cues [29,30,31]. Specifically, ppGpp is upregulated during amino acid starvation, halting replication and activating survival pathways in *E. coli* and *Pseudomonas aeruginosa*. The HokB-SokB type I TA module is activated via ppGpp, inducing persistence [32]. ppGpp also functions as an intermediate to activate obg-mediated persistence. Obg, a GTPase protein involved in DNA replication, triggers HokB, which disrupts membrane potential and reduces metabolic activity, enhancing persistence. Recent studies have further elucidated the role of obg in persister cell formation [33]. Figure 2 illustrates the pathways for ppGpp production and persister formation through HokB–SokB activation.

As referred to by Pacios et al. [34] in their review, (p)ppGpp levels can accumulate in response to a wide range of signals, including oxygen variation, pH downshifts, osmotic shock, temperature shift, or even exposure to darkness. As such, the stringent response is not only involved in responses to environmental stress but is also used in bacterial pathogenesis, host invasion, antibiotic tolerance, and persistence.

### 5.3. Hunker Theory of Persistence

For antibiotics to reach their target, they must first penetrate the cell wall and then bind to the target. Cells that slow down their essential processes (such as growth, metabolism, or antibiotic activation) will be killed more slowly, leading to a state of persistence. This “hunker down” theory of persistence aligns with the finding that persisters are not necessarily slow-growing, as seen in the specialized class of persisters [16]. According to Urabiec et al. [16], not all slow-growing cells are persisters, and not all persisters are slow-growing. Furthermore, while low growth may predispose a cell to enter a state of persistence, it is neither sufficient nor a necessary condition.

### 5.4. SOS Response Connected with Both TA Systems and Efflux Pumps

The SOS response, in conjunction with TA systems or efflux pumps, plays a pivotal role in the formation of persister cells, thereby enhancing bacterial population tolerance to antibiotics. This DNA damage repair system, regulated via LexA and RecA, is essential for bacterial adaptation and the development of antibiotic resistance. Notably, in *E. coli*, the TisB/IstR module is the only known toxin–antitoxin system regulated via the SOS response that directly contributes to persister formation. Additionally, the SOS response is crucial for the formation of antibiotic-resistant biofilms, particularly in bacteria such as *E. coli*, *P. aeruginosa*, *Staphylococcus aureus*, and *Mycobacterium tuberculosis* [35]. Biofilms in dynamic environments generate compounds that induce DNA damage, thereby promoting adaptation and resistance.

### 5.5. Persistence as “Stuff That Happens”

**Figure 3:**
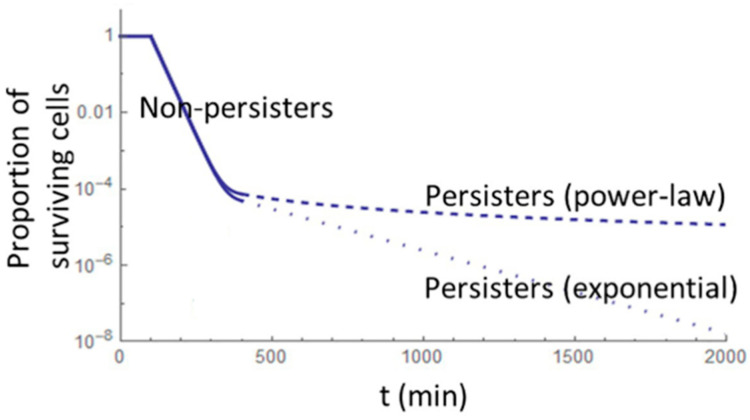
Representation of bacterial population decay in the presence of an antibiotic. The vertical axis represents the proportion of surviving cells, the horizontal axis represents exposer time (min). In this representation, it is possible to see a steep reduction in bacterial cells (non-persisters susceptible to antibiotic activity) in the first 500 min of exposer and the appearance of a power-law-like tail representing persisters survival. Figure adaptation from Rebelo et al. [10].

Type II persisters may also be explained as cells that arise as an inevitable consequence of “errors” during cell cycle and division, which introduce phenotypic heterogeneity. This formation is referred to as “persistence as stuff that happens” (PaSH) [9], asserting that persistence may not be an adaptive trait but, rather, a result of stochastic processes. It is also possible that the different persister classes described above have distinct evolutionary origins. In this scenario, antibiotics do not directly induce the production of persister cells but, rather, “unmask” an already-existing subpopulation in a power-law decay curve (Figure 3) [8]. According to this model, persister cells are not a response to antimicrobial stress but are continuously present within the bacterial population. To test this hypothesis, researchers studied the persistence of *S. aureus* (Newman strain) when exposed to various antibiotics, including ciprofloxacin, gentamicin, vancomycin, and oxacillin, at concentrations ranging from the minimum inhibitory concentration (MIC) to 10 × MIC. These experiments led to the development of a mathematical model to predict persister cell generation and selection. Interestingly, cultures pre-treated with gentamicin or ciprofloxacin exhibited an increased persistence rate when later exposed to other antibiotics. While the researchers acknowledge that antimicrobials can stress populations and lead to cell division arrest (type I persisters), they also suggest that persisters may arise from stochastic errors in cell replication, similar to mutations [8,9,21].

### 5.6. Other Systems and Forms Contributing to Persistence

In the food industry, bacterial persistence extends beyond individual persister cells to include systems and forms that present significant challenges to food safety: biofilms. These structured bacterial communities are embedded in a protective matrix, commonly found on food processing surfaces, where they shield pathogens from cleaning and disinfection procedures [36]. Small colony variants (SCVs), with their slow growth and altered metabolism, can persist in food products and equipment, increasing the risk of contamination and spoilage. SCVs are similar to persisters since they are very hard to grow in the lab and to destroy using conventional methods. The foodborne pathogen *S. aureus*, when in harsh conditions, is known to produce biofilms, but also, these biofilms may harbor persisters, as well as SCVs. SCVs have been identified in several food samples and are described as the survival strategy of *S. aureus* alongside biofilm formation to endure acidic pH environments [35]. In addition, SCVs in *S. aureus* also have a high biofilm-producing capacity contributing to the persistence of this species in food-industry settings [37]. Other known foodborne microorganisms like *Listeria monocytogenes* [38], *Bacillus cereus* [39], or *P. aeruginosa* [40] have also been studied regarding their capacity to produce SCVs and their survival strategies.

L-form bacteria, which lack a cell wall and are resistant to standard antimicrobial treatments, can survive in processed foods or under certain production conditions. It is possible that persister cells, present on *Listeria* enrichment media (LIM), can later form L-form colonies. L-form cells themselves could also be considered a type of persister, as they, unlike their walled counterparts, can grow in the presence of beta-lactam antibiotics. This phenomenon was described by Glover et al. [41] in *E. coli*.

Finally, spores and intracellular persisters are two survival strategies employed by various bacteria, including members of the genus *Salmonella*, *Listeria*, and *Bacillus*, to withstand extreme processing conditions such as heat, drying, and chemical sanitization. These mechanisms contribute to recurring contamination in food systems. While not extensively covered in this review, these forms of persistence have been discussed by Shiqi Liu et al. [42] in the context of their potential inactivation by antimicrobial peptides (AMPs), or by Fu et al. [43] with the discovery of enzymes with lytic activity against spores produced by some *Bacillus* genera. Together, these strategies emphasize the multifaceted nature of bacterial survival and underscore the need for diverse and combined eradication approaches.

Although persistence itself is non-heritable, the frequency of persister cells in a given population is indeed a heritable trait. Several studies have shown that increased exposure to stressors, including antibiotics, leads to a higher frequency of persister cell production and enhanced fitness [6,8].

Table 1 summarizes and associates the different mechanisms of formation discussed above to the different types of persisters for better understanding.

**Table 1:** Different types of persisters and their formation mechanisms.

Type of Persister	Production Stage	Formation Mechanism	References
Type I	Stationary phase	TA systems, SOS response (connected with TA system and efflux pumps), and spores	[15,19,20]
Type II	Continuous growth at slow rate	TA systems, stringent response, and SCVs	[15]
Type III	Induced by specific antibiotics	TA systems, hunker theory, PaSH, and L-form bacteria	[17,18,20]

## 6. Implications of Persister Cells in the Food Industry

Although studies on persister cells specifically related to food safety are limited, their formation mechanisms, survival, and regrowth have been well documented, as described in the previous sections. Persister cells can survive adverse conditions such as heat, cold, and chemical treatments without undergoing genetic mutations. This resilience poses significant challenges to food safety, as these cells can survive sanitation procedures and later regrow, leading to food contamination or spoilage.

The presence of persister cells is particularly worrying when foodborne pathogens are present, namely *Salmonella* spp., *E. coli*, *S. aureus*, *L. monocytogenes*, or *B. cereus*. These microorganisms can form biofilms on food surfaces and equipment, impeding their elimination. Fernandes et al. [44] showed that persister cells from *B. cereus* and *Pseudomonas fluorescens* are able to survive biocide exposure. Similarly, *B. cereus* spores have been found to survive cooking processes in rice dishes and later reactivate, posing further risks to food safety [45]. Additionally, *Bacillus* may also be found in meat products (producer of toxins in temperatures of 17 °C), spices (dry garlic, laurel, or pepper), dairy, salted and smoked fish, bakery products (flour and dough), and canned foods [46].

*L. monocytogenes* is another pathogen known for its persistence under extreme conditions, such as high salt concentrations and low temperatures. Its ability to form biofilms on surfaces in food-processing environments makes it difficult to eradicate, even with aggressive cleaning and disinfection protocols [47,48]. Recent research identified stress survival islets (SSIs) in *L. monocytogenes* that contribute to their ability to survive under various environmental stresses [49]. Moreover, toxin–antitoxin systems in *L. monocytogenes* are overexpressed in response to antibiotic exposure, further complicating efforts to control its persistence [50]. In a recent study, Li et al. [51] also tested the presence of *L. monocytogenes* persisters in a simulated processing plant of leafy green products (fresh fruits and vegetables), as well as packing houses. Their main findings were that *L. monocytogenes* persisters’ formation remained steady in nutrient-rich environment and decreased in a nutrient-poor one. This showed a connection between nutrient availability and persister formation. In addition, chloride treatments were also tested regarding their sanitization properties against *Listeria* persisters. The authors observed that an exposer at a concentration of 100 mg/L over two minutes decreased the number of persister cells. Recently, several reviews have featured the factors that contribute to the persistence of *Listeria* in food processing, as well as strategies and interventions [52,53]. *Listeria* persisters may be found in several foods, such as seafood (frozen shellfish and shrimp), meat or poultry (in frozen meat up to 20 days in lamb, 14 months in pork, and 9 months in beef), sandwich or deli meat, vegetables and greens (up to 600 days), and dairy (more than 4 months), as well as its processing facilities [46].

*S. aureus*, a common commensal bacterium, poses a significant risk in food-processing environments due to its biofilm-forming capabilities and potential for producing enterotoxins that can lead to gastrointestinal intoxication. These bacteria can persist in foods such as frozen meat (for more than a year), milk (up to 4 months), or canned foods, being able to produce toxins in environments until 15% of NaCl [46]. Studies have shown that *S. aureus* persister cells can emerge stochastically during the stationary phase, primarily triggered via ATP depletion [54,55]. This contrasts with *E. coli* and *L. monocytogenes*, for which persister formation is closely linked to toxin–antitoxin modules and the stringent response.

Moreover, *Salmonella* spp. and *Campylobacter jejuni* are notable for their persistence in dry food-processing environments, meat and poultry products, both fresh and frozen (up to 1 year for *Salmonella* and 60 days for *Campylobacter*), vegetables and fruits, dairy products (20 days to 1 year for *Salmonella* and 22 days to 8 months for *Campylobacter,* depending on the dairy product) [46]. Recent studies indicate that *C. jejuni* forms persister cells when exposed to antibiotics such as ciprofloxacin and penicillin G [56,57]. However, research on *C. jejuni* persisters remains limited. Table 2 compiles information on foodborne bacteria and persister cells’ formation (whenever detailed information could be retrieved from previous publications).

**Table 2:** Foodborne bacteria and mechanisms associated with the production of persister cells.

Microorganism	Production Inducers	Persister Cells Development Mechanism	Food Type	References
*L. monocytogenes*	Environmental triggers and/or stressful conditions associated with temperature, NaCl, pH, or the presence of antimicrobials	TA systems Stringent response Biofilms	Food-processing environment, meat, dairy (milk, soft cheese, and butter), leafy greens (vegetables and fruits), seafood, bakery products, and sandwiches	[46,49,50,51,52,53]
*B. cereus*	Heat and desiccation	Spore formation and biofilms	Cooked foods, rice, canned products, salted and smoked fish, milk and dairy, and meat	[45,46]
*S. aureus*	ATP depletion	Stochastically Biofilms	Food-processing environment, fish, seafood, bakery and canned products, eggs, milk, plant-based foods, and meat	[46,54,55]
*P. fluorescens* *P. aeruginosa*	Environmental triggers and/or stressful conditions associated with temperature, NaCl, pH, or the presence of antimicrobials	TA systems Stringent response Biofilms	Dairy, vegetables, meat, and ready-to-eat foods	[44]
*E. coli*	Environmental triggers and/or stressful conditions associated with temperature, NaCl, pH, or the presence of antimicrobials	TA systems Stringent response Biofilms	Cooked meat, vegetables, berries, fruits, milk, and eggs	[46]

The food industry faces a unique challenge in controlling bacterial persistence. Food processing and preservation often involve conditions such as fluctuating salt, temperature, pH, and nutrient availability, which can trigger bacteria to enter a persister state. One major contributor to persister formation is the activation of TA systems. These systems are versatile and respond to various stresses common in food-processing environments, such as nutrient limitation (e.g., during preservation) and high salt concentrations (e.g., in salted or fermented foods). TA systems are also key players in biofilm formation, which further protects persister cells. Another important mechanism is the stringent response, triggered by nutrient scarcity. This response activates stress survival pathways and can be induced during minimal processing or long-term storage. Additionally, the use of sanitizers and disinfectants can inadvertently activate the SOS response, increasing persister formation and antimicrobial tolerance. Finally, the presence of biofilms in food-processing environments further complicates control efforts since these structures provide a protective environment for persister cells and act as reservoirs for spoilage or pathogens, increasing the risk of contamination and foodborne illnesses.

### 6.1. General Preventive Measures

In the EU and in some other areas of the world, food manufacturers must identify and control food safety hazards. The use of strict hygiene protocols, the implementation of standards like ISO 22000—Food Safety Management, and the implementation of Hazard Analysis Critical Control Point (HACCP) framework [58,59] aid in the identification and mitigation of contamination risks, which indirectly includes persister cells. These standards or systems benefit producers and manufacturers, control authorities, retailers, and finally consumers.

### 6.2. Targeted Eradication Approaches

Microbial contamination in the food industry can have devastating economic and social consequences due to equipment malfunction, the cross-contamination of products, spoilage, and the need for the recall of products, ultimately causing foodborne illnesses. The presence of persister cells presents a “silent” challenge to the industry quality control. To address this issue, various prevention and eradication strategies have been proposed, including optimized sanitation practices and targeted interventions. Furthermore, biofilm-forming persisters pose an additional challenge to treatment due to the extracellular polymeric substance (EPS) that impedes antimicrobial penetration, reducing the efficacy of conventional therapies [36].

Previous research [16,60] categorizes persister-eradication strategies into four primary approaches:

(i) The direct killing of dormant persister cells: This involves targeting cellular structures such as the cell wall, the membrane, and DNA. By disrupting membrane potential or altering permeability, persisters become susceptible to antimicrobials. Physical methods, such as heat, UV radiation, and sonication, directly damage cellular structures, complementing this approach. Chemical agents, including surfactants and reactive oxygen species (ROS), can further enhance membrane disruption, while biological methods, such as bacteriophage-derived enzymes, target cell walls with precision.
(ii) Awakening dormant cells: Some approaches aim to “wake” persister cells, making them metabolically active and, therefore, more vulnerable to antibiotics. Metabolic triggers like pyruvate, often used as chemical agents, can effectively induce cellular activity. Physical methods, such as alternating temperatures or pressures, can also provoke metabolic changes. Additionally, biological tools, including certain enzymes or signaling molecules, may assist in reactivating dormant cells.
(iii) Combination therapies: Combining anti-persister agents with conventional antibiotics enhances treatment effectiveness. This diversified approach attacks persisters through multiple mechanisms. Physical methods can act synergistically with chemical antimicrobials, e.g., heat-enhanced antibiotic activity. Similarly, biological methods, such as combining quorum-sensing inhibitors with antibiotics, amplify the impact of chemical treatments.
(iv) Quorum-sensing interference: Targeting quorum-sensing circuits can prevent persister cells from communicating and forming biofilms. Biological strategies, such as enzymes that degrade quorum-sensing molecules or peptides that block receptors, are highly effective and have been described in previous publications [52,53]. Chemical agents can inhibit quorum-sensing molecule synthesis, while physical methods, like ultrasound, may disrupt biofilm structures, indirectly interfering with quorum-sensing pathways [52,53]. This diversified approach attacks persisters through multiple mechanisms improving the chances of success.

The following sections delve into physical, chemical, and biological eradication treatments, exploring their mechanisms of action and their alignment with these targeted strategies to control and eliminate persister cells in the food industry.

### 6.3. Physical Treatments

Numerous physical methods have been developed for the direct elimination of persister cells (see Table 3). These approaches arise from the highly dormant nature of persisters, which impairs the effectiveness of all antimicrobial agents, whether natural or chemically synthesized [60]. Additionally, several studies suggest that specific physical methods may also help prevent the development of antimicrobial tolerance [61,62].

High-temperature processing methods, including pasteurization, sterilization, and thermal treatment, are widely utilized in the food industry due to their effectiveness in inactivating pathogenic and spoilage microorganisms. These processes achieve microbial control through protein denaturation, membrane disruption, and enzyme inactivation, thereby ensuring food safety and extending shelf life [63]. Key advantages of high-temperature treatments include their reliability in pathogen reduction, scalability, and cost-effectiveness. However, limitations such as nutrient degradation and alterations in texture, color, and flavor, as well as high energy consumption, must be considered. The suitability of thermal processing varies across different food matrices [64]. In dairy products, pasteurization effectively controls microbial contamination while preserving nutritional quality, whereas ultra-high-temperature (UHT) treatment extends shelf life but may induce slight flavor modifications [63]. In meat and poultry, high-temperature processing ensures microbiological safety but can lead to protein denaturation and moisture loss, effects that can be mitigated through sous-vide techniques [65]. In fruits and vegetables, blanching and pasteurization inhibit enzymatic spoilage and microbial proliferation, though potential texture softening and nutrient loss remain concerns [66]. For canned and ready-to-eat foods, thermal sterilization ensures commercial sterility and prolonged stability, albeit with potential adverse effects on sensory attributes. In the beverage sector, thermal pasteurization effectively eliminates pathogens but may compromise freshness and color stability, leading to increased interest in milder heat treatments and non-thermal alternatives [67]. Despite these challenges, high-temperature methods remain fundamental to food safety and preservation, with ongoing research focused on optimizing processing conditions to balance microbial control with the retention of quality attributes.

The efficacy of high-temperature methods against persisters can be enhanced through combination approaches. Integrating mild thermal treatments with bacteriocins, enzymatic treatments, or pressure-based methods may improve persister elimination while minimizing adverse effects on food quality. Optimizing thermal conditions to achieve effective inactivation without compromising sensory and nutritional properties remains crucial. Further research is needed to refine these strategies and develop tailored thermal treatments for the improved control of persister cells in food systems.

High hydrostatic pressure (HHP) is recognized for its effectiveness in eliminating vegetative bacterial cells, yet it shows limited efficacy against persister cells, especially endospore-forming variants like *B. cereus* [68]. To address this challenge, a two-pronged approach is necessary: an initial pretreatment designed to induce spore germination. HHP alone may not adequately target persister cells in their dormant state; therefore, inducing spore germination is crucial to making them susceptible to the detrimental effects of HHP. However, it is important to note that, even with this pretreatment, non-germinating spores may persist within the food matrix post-HHP treatment.

To enhance the eradication of persister cells, industrial practices often combine HHP with additional treatments, such as thermal treatments (ranging from 50 °C to 100 °C) and the incorporation of essential oil compounds [69]. For instance, Evelyn et al. [68] demonstrated that increasing HHP (up to 600 MPa) and elevating the temperature from 38 °C to 70 °C significantly improved *B. cereus* spore inactivation in milk.

One key advantage of HHP treatment, compared to traditional high-temperature methods, is that it preserves the organoleptic and nutritional properties of the food matrix (e.g., flavor and vitamin content), which is crucial for consumer satisfaction and maintaining overall food product quality [70]. Additionally, HHP extends shelf life, reduces the need for chemical additives, and ensures uniform processing, regardless of food shape or size. It also meets the growing consumer demand for clean-label products by providing a natural preservation method [71]. Its application in juices, dairy, seafood, meat, ready-to-eat meals, and functional foods enables product innovation while preserving bioactive compounds beneficial to health [72]. HHP is an excellent choice for manufacturers seeking to offer high-quality, fresh-tasting, and minimally processed foods. Its isostatic nature ensures uniform treatment, and in-package processing minimizes contamination risks. Furthermore, HPP is environmentally friendly, as it relies solely on electric energy without generating waste products [73]. However, its feasibility depends on the product type, processing goals, and economic considerations [71].

Despite its many benefits, HHP involves some disadvantages. The high initial cost of equipment makes it less accessible to small-scale producers, limiting its widespread adoption. Additionally, while it effectively inactivates vegetative microorganisms, it has a limited impact on bacterial spores unless they are combined with heat (pressure-assisted thermal sterilization). Residual enzyme activity and dissolved oxygen can lead to enzymatic and oxidative degradation of certain food components. To maintain sensory and nutritional quality, most pressure-treated foods require refrigeration during storage and distribution. Moreover, for HPP to achieve an antimicrobial effect, foods should contain approximately 40% free water, restricting its application to specific products [73].

Therefore, effectively mitigating persister cells, particularly endospores, in the context of food safety requires a multifaceted strategy. By integrating high hydrostatic pressure with spore germination pretreatments and complementary methodologies, a more efficient approach emerges for reducing the resilience of these persistent bacterial phenotypes while simultaneously safeguarding the sensory and nutritional characteristics inherent to the food product.

The use of direct electric current is an effective method for controlling biofilm formation and eliminating persister cells [74]. Niepa et al. [75] investigated the effects of low-level electrochemical current (70 µA/cm^2^) in conjunction with tobramycin (1.5 µg/mL) against *P. aeruginosa* persister cells. This strategy’s success likely stems from the sensitization and increased susceptibility of persister cells to antimicrobials, a process facilitated by the disruption of the cytoplasmic membrane. Following this disruption, persisters were effectively killed through the application of the electrochemical current generated via carbon and stainless-steel electrodes. This dual approach not only enhances the effectiveness of the antimicrobial agent but also demonstrates the potential of electrochemical methods in addressing persister cell challenges in biofilm management.

One of the main advantages of low-level electrochemical current is its non-thermal nature, allowing food to retain its sensory and nutritional quality without the texture, flavor, or nutrient degradation associated with thermal processing. It is also energy-efficient and applicable to both liquid and solid food systems, offering flexibility in food preservation. Additionally, this method can reduce the need for chemical preservatives, aligning with clean-label trends. However, its effectiveness varies based on factors such as food type, microbial species, and treatment duration, with some resistant biofilms requiring prolonged exposure for significant reductions. However, its application requires further optimization to ensure uniform effectiveness without affecting sensory properties. While this method offers a promising non-thermal approach to food preservation, additional research is necessary to refine its application, address safety concerns, and expand its usability across diverse food products.

Additional physical strategies discussed in the literature include the use of magnetic fields and ionizing irradiation [74,75,76]. Thus far, the inactivation of microorganisms using a magnetic pulsed field has shown limited efficacy in preventing bacterial biofilm formation [77]. However, its specific impact on persister cells has not been thoroughly investigated.

In contrast, ionizing irradiation is a well-established method that can damage microbial DNA, contributing to food safety and quality when applied at appropriate doses [76]. The Food and Drug Administration (FDA) has approved irradiation as a technique to control the growth of microorganisms on food surfaces [77]. Although irradiation has been effectively employed to manage bacterial biofilms, its effects on persister cells remain unexplored. Further research into these physical strategies could provide valuable insights into their potential to target persister cells, thereby enhancing food safety protocols in the industry.

In addition to the previously mentioned physical techniques for treating persisters in the food industry, several other methodologies may contribute to preventing the development of foodborne microorganisms. These non-thermal techniques include cold plasma, ultraviolet (UV) light irradiation, high-intensity light pulses (HILP), pulsed electric fields (PEFs), and ultrasound (US) [78]. However, further research is needed to optimize their efficacy related to persisters.

### 6.4. Chemical Treatments

In addition to the previously discussed physical methods, a range of chemical agents can be utilized against microbial cells in both planktonic and biofilm states [46,67,68,69,70]. The efficacy of these agents is influenced by factors such as the specific agent employed, its concentration, and the duration of contact with microorganisms. Following application, the microbial load must be reduced to levels deemed safe for human consumption, a process referred to as sanitization [79]. The proper sanitization of food processing equipment is crucial to prevent cross-contamination between food batches [80].

Chemical changes during food production processes, such as variations in pH and osmotic pressure, can significantly influence the development of persister cells. It is well known that lowering pH below 4.6 inhibits the growth of many spoilage and pathogenic microorganisms [81]. Similarly, increasing osmotic pressure by adding salt (curing) or sugar (syrup preservation) creates a hypertonic environment that causes water loss from microbial cells through osmosis, leading to dehydration and the inhibition of microbial activity [82]. These preservation methods are ideal for fermented and pickled foods, fruit preserves, salted meats, and syrups. Acidification is commonly used in dairy products, canned vegetables, and fermented beverages, while osmotic pressure-based preservation works well for dry-cured meats, salted fish, jams, honey, and candied fruits. These techniques are best for foods requiring mild processing and limited refrigeration, but their effectiveness depends on proper formulation and storage to prevent spoilage via acid- or salt-tolerant microorganisms [83]. Xiong et al. [84] noted that low pH conditions (pH < 4.5) have varying effects on different bacterial species; for instance, persister *E. coli* can produce toxins under low pH conditions. Changes in osmotic pressure also impact cell growth rate, turgor pressure, and transport mechanisms between cells and their extracellular environment. One of the main advantages of using pH and osmotic pressure for food preservation is that these methods are natural and do not require high temperatures or synthetic preservatives, aligning with consumer demand for clean-label products. Acidification not only prevents microbial growth but can also enhance flavor, as seen in fermented foods like yogurt, sauerkraut, and vinegar-based pickles. Osmotic pressure methods such as salting and sugaring are simple and cost-effective, and they have been used for centuries to preserve meats, fish, and fruits. However, extreme acidity can affect taste and texture, making foods less desirable. Some microorganisms, like yeasts and molds, can survive in acidic or high-sugar environments, leading to spoilage if storage is not controlled. High salt or sugar concentrations can also impact the nutritional balance, with excessive intake linked to health issues like hypertension and diabetes. Despite these challenges, adjusting pH and osmotic pressure remains a reliable method for preserving food, ensuring safety and quality while supporting traditional processing practices.

Karki et al. [85] investigated the effects of sodium nitrite, urea, and acidic pH on the survival of *E. coli* persisters. These authors found that urea (4%) and sodium nitrite (80 mM) reduced persister levels below the limit of detection while having minimal effects on overall cell viability, as measured using CFU counts. In contrast, lowering the pH below 4.5 significantly compromised *E. coli* cell viability. Although sodium chloride (NaCl) is commonly used in cell culture media, Karki’s research indicated that concentrations below 8% do not substantially affect the viability or persister levels of *E. coli*. However, elevated NaCl concentrations pose a significant threat to *E. coli* vitality, as demonstrated by the experimental results. The precise mechanisms through which NaCl concentration influences persister formation remain to be elucidated [84].

Among the most widely used biocides in the food industry are oxidizing agents, including halogen-based compounds, peracetic acid (PAA), ozone, and hydrogen peroxide. These agents are particularly effective at inactivating a broad spectrum of microbial cells, both in planktonic form and within biofilms [86]. Chlorine-based solutions such as liquid chlorine, hypochlorites, and chloramines are extensively utilized for their potent antimicrobial properties, while surface-active agents like quaternary ammonium compounds (QACs) disrupt microbial cell membranes [87].

For example, Fernandes et al. [44] examined the antimicrobial activity of two conventional biocides—benzalkonium chloride (BAC) and peracetic acid (PAA)—alongside two emerging biocides—glycolic acid (GA) and glyoxal (GO)—against persisters of *B. cereus* and *P. fluorescens*. These agents can be effectively applied to floors, sewers, and food-industry equipment but should not be used directly on food. The study concluded that persister cells were prevalent within biofilms and survived exposure to critical biocide treatments. Additionally, the descendant planktonic and biofilm populations exhibited properties similar to the original cells. The impact of these biocides on spore-forming bacteria, such as those in the *Clostridium* and *Bacillus* genera, remains uncertain. Poor hygiene, sanitation, and handling practices can facilitate the transfer of biocide-tolerant bacteria to food, underscoring the necessity of combining multiple methods to effectively eliminate hazardous microbes.

Drug combination therapies have been used to target persister cells in medical contexts and could be adapted for food safety purposes. These combinations can include two or more antimicrobial peptides or a mix of peptides and other low-cytotoxicity chemical compounds. Khan et al. [60] provide several examples of anti-persister combinations. However, if these combinations contain antimicrobials or other chemical compounds not approved for use in food-related contexts, their application in the food industry may be limited. Additionally, some researchers have suggested that interfering with the toxin–antitoxin system could induce the metabolite-driven awakening of persisters, allowing them to transition to an antimicrobial-susceptible state [88,89].

Incorporating carbon sources like mannitol and glucose can enhance the sensitivity of persisters to antimicrobial peptides in *E. coli* by increasing their metabolic activity. For instance, exposure to 40 mM of mannitol significantly reduces biofilm viability, improving antimicrobial efficacy by 99.96%. Similarly, 40 mM of glucose mitigates nutrient depletion resulting from heightened metabolic activity, substantially inhibiting persister production without affecting non-dividing cells [90]. Polysulfonic mucopolysaccharides and trehalose (at a concentration of 1%) also deter persister production by preventing protein aggregation and oxidation; however, higher concentrations can stimulate aggregation and persistence formation [91]. Furthermore, low concentrations of betaine and glycerin in the growth medium help prevent protein aggregation and persister formation in *E. coli*, while higher concentrations (1%) of both substances can increase persister levels and protein aggregates. The use of different carbon sources plays an important role in food preservation by influencing microbial stability, water activity, and texture. Glucose is commonly used in high concentrations to create a hypertonic environment that inhibits microbial growth, making it effective in preserving foods like jams, syrups, and candied fruits. It lowers water activity, reduces spoilage, and enhances sweetness and texture [92]. Mannitol, a sugar alcohol, is less metabolized by many microorganisms, making it stable in certain food systems and beneficial in diabetic-friendly, sugar-free, and dental-friendly products [93]. However, their effectiveness depends on food type and microbial strains, and their use has limitations. High concentrations of glucose can lead to excessive sweetness, contributing to health concerns like obesity and diabetes, and may promote Maillard reactions, which can be undesirable in some foods. Mannitol can cause digestive discomfort if consumed in large amounts due to its laxative effect, and certain microorganisms, such as osmophilic yeasts and molds, can still grow in high-sugar environments. Glucose is well suited for traditional preservation methods in confectionery, dried fruits, and baked goods, while mannitol is ideal for sugar-free products, functional foods, and pharmaceuticals. The suitability of both compounds depends on the desired sensory attributes, nutritional considerations, and regulatory limitations regarding sugar intake and sugar alcohols.

### 6.5. Biological Treatments

In recent years, interest has grown in using natural antimicrobials, also called green biocides, to combat persister cells [94,95]. These compounds are typically safe for human consumption and do not adversely affect food quality, prompting increased research into their potential for eliminating persisters in the food industry. Green biocides are produced by various organisms, including plants, animals, bacteria, algae, and fungi, and they exhibit antibacterial activity against primary foodborne pathogens, showing significant promise for application in the food sector. However, challenges such as high volatility, residual taste, and degradation under harsh processing conditions have been noted as primary obstacles [95].

Plant-derived antimicrobials offer a promising and eco-friendly strategy for addressing persister cells in the food industry. Aromatic and medicinal plants serve as the primary source of these antimicrobials, particularly in the extraction of essential oils. Several studies have documented their effectiveness in natural food preservation and quality enhancement [96,97,98]. In addition to essential oils, other plant extracts rich in phenolic and bioactive components exhibit antibacterial and antibiofilm activity against various spoilage and pathogenic microorganisms [99]. Utilizing plant-derived antimicrobials presents an effective and sustainable approach to enhancing food safety and extending product shelf life while aligning with the demand for natural and environmentally friendly food preservation methods. They also contribute to improved flavor, aroma, and antioxidant stability in food [97]. Certain plant extracts are commonly used to delay lipid oxidation in meat and dairy products [98]. However, their strong flavors and aromas can alter sensory properties, limiting their application. Their effectiveness depends on interactions within the food matrix, requiring optimization for each specific use. In some cases, high concentrations may be needed for efficacy, increasing production costs. Additionally, regulatory restrictions may apply to certain plant-derived compounds. Plant extracts and essential oils are particularly suitable for meat, dairy, baked goods, and minimally processed foods, especially in natural and organic products. Their successful application depends on balancing antimicrobial effectiveness with sensory acceptability and regulatory compliance.

Nevertheless, previous studies have emphasized the need to correlate the effects of essential oils and extracts with their composition, concentration, and the bacterial strains involved. While research specifically targeting the use of essential oils to eliminate persisters in food is currently lacking, this area offers a compelling opportunity for future investigation. Notably, Lu et al. [100] found that combining carvacrol with blue light can synergistically eliminate a broad spectrum of bacteria. This phenomenon occurs as carvacrol, a phenolic monoterpenoid found in various essential oils and aromatic plants, may undergo oxidation by reactive oxygen species (ROS) generated from the excitation of endogenous porphyrin-like substances under blue light. When 50 μL of carvacrol was combined with 450 nm blue light, bacterial colony-forming units were reduced by up to 7.5 log10 within just 30 min. While this method is currently more prevalent in clinical applications, existing photodynamic sterilization technologies in the food industry leverage light to activate photosensitizers, producing active oxygen species to eliminate foodborne pathogens. In the future, this technology may be harnessed alongside photosensitizers to effectively eradicate persisters of foodborne pathogens in food products [84].

The most commonly used green biocides for controlling persisters in the food industry include bacteriocins and bacteriophages. Lactic acid and natural antimicrobial peptides (NAMPs), such as bacteriocins and bacteriolysins produced by lactic acid bacteria (LAB), can enhance the quality and safety of fermented foods by inhibiting pathogen multiplication [101,102]. Unlike most antimicrobials that target specific metabolic processes within bacterial cells, NAMPs interact directly with bacterial membranes and can also influence the host’s immune system during infections. Furthermore, several NAMPs can interact with intracellular targets in bacterial cells [103]. Overall, bacteriocins are natural, non-toxic antimicrobial peptides that selectively target harmful bacteria without affecting beneficial microflora, effectively extending shelf life while preserving food quality and safety. However, their activity is limited to specific bacteria, often requiring combination with other preservatives for broader protection. Some bacteriocins lose effectiveness in complex food matrices or under high temperatures, while production costs and regulatory requirements may limit widespread use. Despite these challenges, bacteriocins are particularly suitable for dairy, meat, and minimally processed foods, aligning with clean-label and natural food preservation strategies while maintaining sensory and nutritional properties.

Some bacteriocins, such as Nisin and Pediocin, are commercially available and can be utilized in the food industry [102]. Henderson et al. [104] demonstrated that nisin effectively controls *L. monocytogenes* in food by inducing pore formation in the cell membrane and inhibiting cell wall synthesis. Additionally, Gut et al. [105] found that nisin hampers the growth of *Bacillus* spores by disrupting the endospore membrane. Class IIa Pediocins primarily prevent the invasion of Gram-positive bacteria, such as *L. monocytogenes*, by targeting bacterial mannose phosphotransferase [106]. Rishi et al. [107] further confirmed that 1 μg/mL of nisin, combined with 200 μg/mL of ampicillin and 25 mM of mannitol, effectively eliminates *Salmonella* persister cells. This highlights that the most effective approach to eliminating persisters may involve a combination of different methodologies and/or agents.

Lactoferrin, chitosan, and lysozyme are the most well-studied animal-origin antimicrobials, with lactoferrin being a milk glycoprotein, chitosan a biopolymer from the exoskeletons of crustaceans and arthropods, and lysozyme an antimicrobial enzyme found in eggs and milk [95]. However, there is currently no data available regarding the effects of these plant- and animal-derived antimicrobials on persister cells, making this an intriguing area for further research.

Bacteriophages, which are viruses that infect and replicate within bacterial cells, ultimately leading to bacterial death, have shown potential as effective bacteriolytic agents, especially in cases where antimicrobial agents have failed to eliminate persisters [108,109,110]. Their antimicrobial activity is harmless to humans, animals, and plants due to their specific targeting of prokaryotic cells, making phage therapy an attractive alternative to antibiotics [108,111,112]. Additionally, their application as antibiofilm agents has led to commercial uses, such as *Listeria* phage P100, marketed as Listek P100, which is utilized to eliminate biofilms in processed meat products and factory workshops. This phage has received approval in the United States from the Department of Agriculture, granting it Generally Recognized As Safe (GRAS) status as a biological agent [113,114]. Comprehensive reviews by Cacciatore et al. [95] detail other phage-based commercial products, their application methods, and the types of food and surfaces tested. They indicated that bacteriophages are particularly suitable for raw meats, dairy, fresh produce, and ready-to-eat foods, making them an effective natural alternative in organic and minimally processed food products where bacterial contamination is a concern. However, when employing bacteriophages to control foodborne bacteria in food production facilities and processed foods, it is crucial to consider how environmental conditions affect phage stability. These conditions can also influence the physiological state of bacteria, thereby impacting on the interaction between host and virus, as well as the efficacy of phages in reducing bacterial populations [113]. Overall, bacteriophages offer a highly specific and natural approach to food preservation by targeting harmful bacteria without affecting beneficial microflora or food properties. They reduce the need for chemical preservatives and antibiotics while maintaining the taste, texture, and nutritional value of food. However, their specificity can be a limitation, as they are effective only against certain bacteria, requiring tailored formulations. Bacterial resistance may develop over time, necessitating phage cocktails for sustained effectiveness, and environmental factors such as pH, temperature, and food composition can influence their activity. Additionally, regulatory approval and consumer acceptance remain challenges.

Recent advances in recombinant technology have increased interest in using phage proteins as therapeutic agents with specific and effective persister-killing activity. This strategy involves producing phage enzymes and applying them exogenously to target settings, where they specifically target bacterial cell walls, leading to lysis [111]. Phage proteins have shown high effectiveness against persisters in Gram-positive bacteria [111,115]. However, additional strategies for eliminating Gram-negative persisters require further exploration.

Notable research in this area includes studies by Briers et al. [116] and Defraine et al. [117], who conjugated the KZ144 endolysin with the lipopolysaccharide-disrupting SMAP-29 peptide to create the chimeric product Artilysin Art-175. This product demonstrated high effectiveness against Gram-negative bacteria, such as *P. aeruginosa* and *Acinetobacter baumannii*, without inducing cross-resistance [118]. Despite these advancements, most research has been focused on medical applications, indicating a need for further investigation into potential uses in the food industry.

In addition to phage enzymes, some researchers propose using enzymes as “green chemicals” or in combination with biocides for biofilm removal, given their ability to degrade the extracellular polymeric substance (EPS) network of biofilms [119]. Four enzyme types are particularly relevant for biofilm removal: (i) anti-quorum-sensing (QS) enzymes, (ii) oxidative enzymes, (iii) polysaccharide-degrading enzymes, and (iv) proteolytic enzymes [120]. Anti-QS enzymes effectively inhibit the growth of pathogenic bacteria that rely on quorum sensing (QS) for virulence and resistance [121]. Their primary advantage lies in disrupting bacterial communication without directly killing the bacteria, reducing the risk of resistance development. Additionally, they help maintain the balance of beneficial microbes, making them particularly suitable for probiotic foods [122]. However, their effectiveness is confined to bacterial species that depend on QS, and their activity can be influenced by the composition of the food matrix. Furthermore, commercial availability remains limited, which may hinder their widespread application. These enzymes are especially beneficial in fermented foods, dairy products, and meats, where bacterial biofilms contribute to spoilage or contamination. Additionally, probiotic bacteria have been shown to play a role in inhibiting QS mechanisms in foodborne pathogenic bacteria, offering further potential for food preservation [122].

Oxidative enzymes catalyze oxidation reactions that significantly affect the flavor, color, and nutritional quality of food products. These enzymes are widely employed to control oxidation in fats and oils, preventing rancidity, as well as in fruits and vegetables to mitigate enzymatic browning. Their primary effectiveness lies in extending shelf life by slowing oxidative processes, thereby enhancing food stability in terms of flavor and color retention. Oxidative enzymes are particularly valuable in preserving the freshness of cut fruits and vegetables. However, their application requires careful regulation, as excessive oxidation may negatively impact food quality. Moreover, the presence of oxygen can limit the effectiveness of oxidative enzymes, and they are susceptible to denaturation via heat or other processing techniques [123].

Polysaccharide-degrading enzymes are utilized in food processing to modify texture, enhance digestibility, and regulate gelling properties in products such as jams, fruit juices, and sauces [124]. These enzymes also play a crucial role in processing plant-based foods, improving texture and mouthfeel. However, excessive use of these enzymes can lead to undesirable changes in texture, particularly in food products requiring a firm structure, such as jams or jellies. The enzymatic breakdown of polysaccharides may result in excessive softening, negatively affecting product consistency. According to Dai et al. [124], fermentative starters, including yeast and lactic acid bacteria, can influence the performance of polysaccharide-degrading enzymes in foods, contributing to a stable and controlled fermentation process. This, in turn, facilitates the production of safe, flavorful, and nutritious fermented foods.

Proteolytic enzymes are extensively used in food processing for applications such as marination, tenderization, and the production of protein hydrolysates. They also play a significant role in reducing spoilage by degrading microbial proteins, thereby enhancing food safety [125]. However, excessive application of these enzymes can lead to overly soft textures, necessitating the precise control of processing conditions such as temperature and pH to prevent undesirable changes. These enzymes are essential in the processing of meat, dairy, and plant-based proteins, where they contribute to improving texture, enhancing functional properties, and ensuring overall product quality.

The application of enzymes for biofilm control and the elimination of persisters remains limited due to high costs and restricted commercial availability. Additionally, several environmental factors present in the food industry (such as temperature, pH, substrate, food residues, and various types of food processing surfaces) can significantly interfere with the activity, stability, and efficiency of these enzymes. The effectiveness of enzymatic treatments is contingent on the specific preservation goals, the food matrix, and the processing conditions. As such, enzymes represent valuable tools for achieving natural, clean-label food preservation. By gaining a deeper understanding of the distinct roles played by each enzyme, food producers can optimize preservation strategies, thereby maintaining the desired quality, safety, and shelf life of food products.

Additionally, some physical methods can influence enzymatic activity in meat, as described in detail by Abril et al. [125]. Briefly, HHP alters the meat matrix, disrupting cell structures and enhancing enzymatic activity while particularly improving tenderness. It preserves meat color by boosting enzymatic reactions, such as MetMb reductase activity, and facilitates proteolysis and lipolysis during aging and curing, contributing to sensory attributes. Ultrasound enhances enzymatic activity by inducing cavitation, which improves enzyme binding and reaction rates. Moderate-intensity US increases tenderness by modifying cell membranes and promoting the release of myofibrillary proteins. It also enhances color stability in dry-cured meats by accelerating ZnPP formation and preserving meat color through MetMb reductase activation. Electrical stimulation induces electroporation, releasing enzymes and substrates and activating calpain enzymes, which improve meat tenderness. It enhances protein breakdown and contributes to meat quality, with potential benefits in protein extraction and enzymatic activity during meat processing and aging.

Recently, researchers have explored the potential use of nanostructured antimicrobials as alternative disinfectants. The most studied nanocarriers in food systems include nanoemulsions, nanoliposomes, polymer nanoparticles, and nanofibers [95,126,127]. However, their effectiveness in controlling the development of persisters remains to be investigated.

All the abovementioned information on persister eradication is summarized in Table 3.

**Table 3:** Physical, chemical, and biological methods for the control and/or elimination of persister cells.

Treatments	Subtype	Target	Mechanism of Action	Practical Application	Advantages	Disadvantages	References
Physical	High-pressure processing (HPP)	Vegetative cells, biofilms	Disrupts cell walls, membranes, and biofilm matrix	Used in meat, juices, and dairy products seafood, ready-to-eat meals, and functional foods	Preserves the organoleptic and nutritional properties of the food matrix	High initial cost of equipment and limited impact on bacterial spores	[68,71,73]
	Steam sterilization	Spores	Denatures proteins and destroys spore core structures	Sterilization of canned foods, equipment, and packaging	Highly effective in eliminating bacteria, viruses, and spores, utilizing water as the primary sterilizing agent	Can alter texture and flavor, not suitable for dry, powdery, or heat-sensitive products, risk of overcooking	[128,129]
	UV radiation	Vegetative cells, biofilms	Induces DNA damage and ROS generation	Sanitization of surfaces, water treatment, and packaging sterilization, fruits, vegetables, meat, fish, dairy, and cereal products	Ideal for heat-sensitive foods, preserve texture, flavor, and nutritional value, extending shelf life and reducing spoilage	Only effective for surface sterilization since it does not penetrate deep into solid or opaque foods; prolonged exposure and high doses can degrade certain nutrients	[61,129,130]
	Pulsed electric fields (PEFs)	Vegetative cells	Disrupts membranes and electroporates cells	Applied in liquid foods like juices and soups without altering sensory properties	Zero adverse effects on the nutritional value and sensory properties of food materials	Less effective on solid or complex structures and does not inactivate bacterial spores	[78]
	Ultrasonic waves	Biofilms	Cavitation effect disrupts biofilm matrix and detaches cells from surfaces	Cleaning of processing lines and utensils in food production facilities; suitable for fruits, vegetables, meat, fish, dairy, cereal, and emulsified products	Helps retain the sensory and nutritional qualities of food, effectively inactivating bacteria, yeasts, and mold sand improving the efficiency of food emulsification and homogenization	Solid and complex foods are less responsive compared to liquids; ultrasonic equipment can be costly	[78,129]
	UV-C light emitting diodes (LEDs)	Vegetative and biofilms	Induces DNA damage and ROS generation	Fresh fruit and vegetables, washing water, salad leaves, and stainless-steel surfaces	Sustainability, longer lifetimes, lower costs, reduced energy consumption, and minimal maintenance, wavelength diversity	Limited penetration, effectiveness restricted to surfaces, potential food quality degradation, and reduced efficiency on irregular surfaces	[131]
Chemical	Acidic solutions (e.g., lactic acid and acetic acid)	Vegetative cells, biofilms	Lowers pH, disrupting metabolic activity and biofilm stability	Surface decontamination and addition to marinades, fermented, and pickled foods	Extending shelf life, enhancing flavor, and being cost-effective, safe, and easy to use	May alter taste, be corrosive to equipment, cause nutrient loss, have limited effectiveness on certain microbes, or require regulatory compliance	[85]
	Chlorine dioxide	Vegetative cells, biofilms	Oxidative stress damages biofilm matrix and cellular components	Sanitization of processing equipment and water; washing fruits and vegetables	Leaves no harmful residues, does not produce the strong odor, neither produces toxic by-products nor does alters the nutritive and organoleptic qualities of food products, and is effective over a wide pH range (pH 3–8)	Toxic and explosive at high concentrations, can cause health risks; produce surface properties can affect ClO_2_ accessibility to microbes, residual moisture after the water rinsing can promote microbial growth, and not suitable for dried foods	[86,87,132]
	Hydrogen peroxide	Spores, biofilms	Disrupts spore coat and biofilm structure through oxidative damage	Used in food-contact surfaces and packaging sterilization	Highly versatile with no toxic residues	Unstable and decomposes upon standing, agitation, and exposure to light or heating	[86]
	Enzymatic detergents (e.g., proteases and DNases)	Biofilms	Degrades biofilm matrix by breaking down proteins and extracellular DNA	Applied in cleaning protocols for stubborn biofilm removal in drains and equipment	Rich variety, ability to function under various industrial and even extreme conditions (such as high temperatures), offer targeted, effective, and environmentally friendly cleaning solutions	Require careful handling and proper conditions for effectiveness and can be more costly than conventional chemical detergents	[133]
	Peracetic acid	Vegetative cells, biofilms	Oxidative stress damages cellular components	Sanitizing surfaces and utensils	Highly effective, fast-acting sanitizer with strong antimicrobial properties	Corrosive nature, potential irritants, and short shelf life	[134]
Biological	Probiotics (e.g., *Lactobacillus* spp.)	Vegetative cells	Compete for nutrients and produce antimicrobial compounds	Used in fermented foods and meat and dairy products to prevent pathogen establishment	Enhancing gut health, improving food quality, and extending shelf life	Stability, regulatory approval, and individual variability	[135]
	Antimicrobial peptides (AMPs)	Vegetative cells	Disrupts cell membranes and inhibits growth	Inclusion in food coatings or processing liquids for enhanced safety	A natural and effective way to enhance food safety, extend shelf life, and prevent microbial contamination	Stability, cost, regulatory approval, and potential resistance	[136,137]
	Bacteriophages	Vegetative cells and biofilms	Specifically lyses targeted bacteria and biofilms	Biofilm removal on surfaces and equipment; targeted pathogen elimination in ready-to-eat products	Highly specific and natural approach, reduce the need for chemical preservatives and antibiotics while maintaining the taste, texture, and nutritional value of food	Effective only against certain bacteria, bacterial resistance may develop over time, need for regulatory approval	[111,115,138,139]
	Bacteriocins (e.g., nisin)	Spores, vegetative cells	Inhibits spore germination and vegetative cell growth	Applied in cheese, canned foods, and vacuum-packed products	Natural and non-toxic	Limited activity to specific bacteria, lose effectiveness in complex food matrices or under high temperatures, and high production costs	[137,140]
	Spore lytic enzymes	Spores	Breaks down spore coats and weakens spore resistance mechanisms	Used in high-risk food products to control spore-forming pathogens	Powerful, biological method for improving food safety and extending shelf life by targeting spore-forming pathogens	Careful consideration of their specificity, cost, stability, and regulatory hurdles	[43]

### 6.6. Implementation of Sanitation Techniques Recommendations

When implementing eradication methods for persister cells in the food industry, it is crucial to consider an integrated approach. Combining multiple methods, such as pulsed electric fields (PEFs) with enzymatic cleaning, can enhance effectiveness by targeting different microbial structures; this strategy has been suggested as a preservation treatment for liquid food products (juices or milk-based) [112]. This strategy is particularly useful for addressing biofilms and spores simultaneously. A multi-step sanitation process, where physical, chemical, and biological methods are applied sequentially or synergistically, depending on the food type and processing environment, can be highly effective. Additionally, the application of eradication methods should be tailored to the specific food matrix. For instance, non-thermal food processing methods like UV radiation and high-pressure processing (HPP) are ideal for liquid foods [113], while steam sterilization and chemical disinfectants are commonly used for solid food products. The intensity and duration of treatments must be adjusted based on the food composition, as some methods may alter the sensory or nutritional qualities of sensitive products, such as dairy and juices.

Nonetheless, before adopting any of these methods, it is essential to consider regulatory compliance. Certain physical, chemical, and biological methods, including UV radiation, chlorine dioxide, bacteriophages, and antimicrobial peptides, may require regulatory approval from food safety authorities before they can be used in food production. It is important to check local and international regulations, such as those from the FDA or EFSA (depending on the industry’s geographical location) to ensure full compliance with food safety standards. Methods like probiotics and bacteriocins (e.g., nisin) are typically approved for food use, but only at specific concentrations; nisin, for instance, according to FAO/WHO Codex Committee in milk and milk-derived products, is used at 12.5 mg of pure nisin per kg [109]. Similarly, other antimicrobial peptides and enzymatic treatments may need documentation or approval, depending on their mode of action and concentration limits in food or food packaging [105]. Hence, it is advisable to consult local food safety and health agencies to ensure the safe and legal use of any new technology, particularly when applying advanced methods like HPP or PEF, which may still be subject to evolving regulatory standards. Once eradication strategies are implemented, it is essential to conduct routine microbial testing to validate their effectiveness. This ensures that the target pathogens, including persister cells, are effectively reduced and do not regrow under standard storage and handling conditions. Documentation and record-keeping for sanitation procedures, treatment conditions, and regulatory approvals are vital for traceability and to provide proof of compliance during audits and inspections [50].

Scalability and cost considerations also play a key role in the implementation of eradication methods. It is important to assess the scalability of chosen methods for specific production scales, whether for small-scale artisanal food production or large industrial operations. Additionally, a cost–benefit analysis should be carried out to determine the financial viability of implementing new technologies, as some methods may require significant investment in equipment or staff training. However, methods such as UV radiation and antimicrobial peptides might offer cost-effective alternatives, depending on the specific context [114].

Lastly, ensuring the safety of employees is critical. Staff should be properly trained in the safe handling of eradication methods, especially when dealing with hazardous chemicals or high-energy equipment. Safety protocols should be provided for the handling of chemicals, pressurized systems, and biological agents. By addressing these considerations, the implementation of persister cell eradication strategies can be both effective and compliant with food safety regulations.

## 7. Conclusions

In conclusion, bacterial persisters pose a significant challenge to food safety and quality in industrial environments. These cells, due to their dormant and metabolically inactive state, survive standard antimicrobial treatments, including disinfectants and preservatives. Once conditions become favorable, persisters can resume active growth, leading to potential contamination and spoilage. This resilience underscores the need for comprehensive control strategies to manage persister-related risks in food production.

While persister formation is generally categorized into type I (stationary phase-related), type II (stochastically driven via toxin–antitoxin systems), and type III, the impact of these cells on food safety and shelf life has only recently gained attention. Although extensive research has focused on clinical settings, understanding the implications of persisters in food production is crucial for safeguarding product quality and ensuring safety over extended shelf lives.

Effectively addressing persister cells requires a multifaceted approach. The most promising methods for targeting specific pathogens in various food matrices depend on the pathogen type and food matrix characteristics. High-pressure treatments and thermal processing effectively inactivate pathogens, particularly in liquid and semi-solid matrices where uniform pressure and heat distribution are achievable, though high-temperature methods’ application may be limited by food composition and sensory attributes. Chemical treatments, while effective, are primarily restricted to food-contact surfaces due to safety concerns. Green biocides, bacteriocins, and antimicrobial peptides offer safer alternatives, providing targeted activity against persisters while preserving food quality, making them particularly suitable for minimally processed foods, dairy products, and fermented foods where microbial balance is crucial. Emerging technologies such as bacteriophages, enzymes, and nanostructured antimicrobials hold significant potential for pathogen control, with bacteriophages offering specificity against bacterial pathogens in raw meats and ready-to-eat products, quorum-sensing inhibitors aiding biofilm disruption in fresh and processed foods, and nanostructured antimicrobials ensuring enhanced stability and controlled release of active compounds for packaging and food surfaces. While these approaches present promising opportunities, further research is needed to assess their efficacy, regulatory acceptance, and scalability in complex food matrices.

Additionally, a deeper exploration of the feasibility of these strategies in different food production settings is essential. Future studies should evaluate their cost-effectiveness, consumer acceptance, and environmental impact to ensure practical implementation. Furthermore, understanding the potential for persister cell adaptation to these methods over time remains a critical research gap. Developing predictive models to assess persistence risks under varying food processing conditions could enhance preventive strategies and optimize control measures.

Overall, despite the aforementioned advancements, key research gaps remain. The precise molecular mechanisms governing persister formation in food environments are not yet fully elucidated, particularly under different processing conditions. Further studies are needed to identify specific genetic and environmental triggers that influence persister cell survival in food systems. Additionally, the long-term effects of emerging antimicrobial strategies, including their potential impact on microbial communities and resistance development, require in-depth investigation.

Interdisciplinary collaboration will be crucial in addressing these knowledge gaps. Integrating expertise from microbiology, food technology, bioinformatics, and material science may accelerate the development of innovative solutions. Research efforts should also prioritize the creation of standardized protocols to assess the effectiveness of eradication strategies, ensuring reproducibility and facilitating regulatory approval.

Future technological progress should focus on the integration of multiple eradication strategies, combining physical, chemical, and biological methods to maximize efficacy while ensuring food safety and quality. Advances in high-throughput screening and machine learning may help predict persister formation patterns, allowing for more proactive intervention strategies. Moreover, novel food-safe antimicrobial agents, including next-generation bacteriophages and enzyme-based treatments, require optimization for large-scale applications.

As the food industry continues to deepen its understanding of persister biology, there is an opportunity to implement integrated sanitation and eradication strategies that ensure both safety and quality. Expanding research on real-world applications, scaling up laboratory findings to industrial settings, and addressing potential regulatory barriers will be crucial steps in transforming theoretical approaches into practical solutions. Interdisciplinary research, combining microbiology, material science, and food technology, will be crucial to developing innovative solutions that align with regulatory requirements and consumer preferences. Future innovations should focus on developing multi-targeted approaches that align with food production standards and maintain acceptable sensory qualities. By adopting these advanced strategies, the industry will not only enhance public health protections but also improve food sustainability and quality, ensuring a safer and more resilient food supply for the future.

## Data Availability

No new data were created or analyzed in this study. Data sharing is not applicable to this article.

## References

[1] Ayrapetyan M., Williams T.C., Baxter R., Oliver J.D. (2015). Viable but Nonculturable and Persister Cells Coexist Stochastically and Are Induced by Human Serum. Infect. Immun..

[2] Balaban N. (2011). Persistence: Mechanisms for Triggering and Enhancing Phenotypic Variability. Curr. Opin. Genet. Dev..

[3] Lewis K. (2007). Persister Cells, Dormancy and Infectious Disease. Nat. Rev. Microbiol..

[4] Wood T.K., Knabel S.J., Kwan B.W. (2013). Bacterial Persister Cell Formation and Dormancy. Appl. Environ. Microbiol..

[5] Bigger J. (1944). Treatment of staphylococcal infections with penicillin by intermittent sterilisation. Lancet.

[6] Moyed H.S., Bertrand K.P. (1983). HipA, a Newly Recognized Gene of *Escherichia coli* K-12 That Affects Frequency of Persistence after Inhibition of Murein Synthesis. J. Bacteriol..

[7] Moyed H.S., Broderick S.H. (1986). Molecular Cloning and Expression of HipA, a Gene of *Escherichia coli* K-12 That Affects Frequency of Persistence after Inhibition of Murein Synthesis. J. Bacteriol..

[8] Johnson P.J.T., Levin B.R. (2013). Pharmacodynamics, Population Dynamics, and the Evolution of Persistence in *Staphylococcus aureus*. PLoS Genet..

[9] Levin B.R., Concepción-Acevedo J., Udekwu K.I. (2014). Persistence: A Copacetic and Parsimonious Hypothesis for the Existence of Non-Inherited Resistance to Antibiotics. Curr. Opin. Microbiol..

[10] Rebelo J.S., Domingues C.P.F., Monteiro F., Nogueira T., Dionisio F. (2021). Bacterial Persistence Is Essential for Susceptible Cell Survival in Indirect Resistance, Mainly for Lower Cell Densities. PLoS ONE.

[11] Şimşek E., Kim M. (2019). Power-Law Tail in Lag Time Distribution Underlies Bacterial Persistence. Proc. Natl. Acad. Sci. USA.

[12] Blattman S.B., Jiang W., McGarrigle E.R., Liu M., Oikonomou P., Tavazoie S. (2024). Identification and Genetic Dissection of Convergent Persister Cell States.

[13] van den Bergh B., Fauvart M., Michiels J. (2017). Formation, Physiology, Ecology, Evolution and Clinical Importance of Bacterial Persisters. FEMS Microbiol. Rev..

[14] Balaban N.Q., Helaine S., Lewis K., Ackermann M., Aldridge B., Andersson D.I., Brynildsen M.P., Bumann D., Camilli A., Collins J.J. (2019). Definitions and Guidelines for Research on Antibiotic Persistence. Nat. Rev. Microbiol..

[15] Balaban N.Q., Merrin J., Chait R., Kowalik L., Leibler S. (2004). Bacterial Persistence as a Phenotypic Switch. Science.

[16] Urbaniec J., Xu Y., Hu Y., Hingley-Wilson S., McFadden J. (2022). Phenotypic Heterogeneity in Persisters: A Novel “hunker” Theory of Persistence. FEMS Microbiol. Rev..

[17] Wakamoto Y., Dhar N., Chait R., Schneider K., Signorino-Gelo F., Leibler S., McKinney J.D. (2013). Dynamic Persistence of Antibiotic-Stressed Mycobacteria. Science.

[18] Goormaghtigh F., Van Melderen L. (2019). Single-Cell Imaging and Characterization of *Escherichia coli* Persister Cells to Ofloxacin in Exponential Cultures. Sci. Adv..

[19] Gefen O., Balaban N.Q. (2009). The Importance of Being Persistent: Heterogeneity of Bacterial Populations under Antibiotic Stress: Review Article. FEMS Microbiol. Rev..

[20] Dörr T., Vulić M., Lewis K. (2010). Ciprofloxacin Causes Persister Formation by Inducing the TisB Toxin in *Escherichia coli*. PLoS Biol..

[21] Trastoy R., Manso T., Fernández-García L., Blasco L., Ambroa A., Pérez del Molino M.L., Bou G., García-Contreras R., Wood T.K., Tomás M. (2018). Mechanisms of Bacterial Tolerance and Persistence in the Gastrointestinal and Respiratory Environments. Clin. Microbiol. Rev..

[22] Kim Y., Wood T.K. (2010). Toxins Hha and CspD and Small RNA Regulator Hfq Are Involved in Persister Cell Formation through MqsR in *Escherichia coli*. Biochem. Biophys. Res. Commun..

[23] Guglielmini J., Van Melderen L. (2011). Bacterial Toxin-Antitoxin Systems: Translation Inhibitors Everywhere. Mob. Genet. Elem..

[24] Song S., Wood T.K. (2020). A Primary Physiological Role of Toxin/Antitoxin Systems Is Phage Inhibition. Front. Microbiol..

[25] Pizzolato-Cezar L.R., Spira B., Machini M.T. (2023). Bacterial Toxin-Antitoxin Systems: Novel Insights on Toxin Activation across Populations and Experimental Shortcomings. Curr. Res. Microb. Sci..

[26] Harms A., Brodersen D.E., Mitarai N., Gerdes K. (2018). Toxins, Targets, and Triggers: An Overview of Toxin-Antitoxin Biology. Mol. Cell.

[27] Zhang S.-P., Wang Q., Quan S.-W., Yu X.-Q., Wang Y., Guo D.-D., Peng L., Feng H.-Y., He Y.-X. (2020). Type II Toxin–Antitoxin System in Bacteria: Activation, Function, and Mode of Action. Biophys. Rep..

[28] Paul P., Sahu B.R., Suar M. (2019). Plausible Role of Bacterial Toxin–Antitoxin System in Persister Cell Formation and Elimination. Mol. Oral Microbiol..

[29] Giramma C.N., DeFoer M.B., Wang J.D. (2021). The Alarmone (p)PpGpp Regulates Primer Extension by Bacterial Primase. J. Mol. Biol..

[30] Song S., Wood T.K. (2020). Combatting Persister Cells With Substituted Indoles. Front. Microbiol..

[31] Chowdhury N., Kwan B.W., Wood T.K. (2016). Persistence Increases in the Absence of the Alarmone Guanosine Tetraphosphate by Reducing Cell Growth. Sci. Rep..

[32] Verstraeten N., Knapen W.J., Kint C.I., Liebens V., Van den Bergh B., Dewachter L., Michiels J.E., Fu Q., David C.C., Fierro A.C. (2015). Obg and Membrane Depolarization Are Part of a Microbial Bet-Hedging Strategy That Leads to Antibiotic Tolerance. Mol. Cell.

[33] Verstraeten N., Gkekas S., Kint C.I., Deckers B., Van den Bergh B., Herpels P., Louwagie E., Knapen W., Wilmaerts D., Dewachter L. (2019). Biochemical Determinants of ObgE-Mediated Persistence. Mol. Microbiol..

[34] Pacios O., Blasco L., Bleriot I., Fernandez-Garcia L., Ambroa A., López M., Bou G., Cantón R., Garcia-Contreras R., Wood T.K. (2020). (P)PpGpp and Its Role in Bacterial Persistence: New Challenges. Antimicrob. Agents Chemother..

[35] Hussain Chan M.W., Mirani Z.A., Khan M.N., Ali A., Khan A.B., Asadullah, Rauf N. (2021). Isolation and Characterization of Small Colony Variants of *Staphylococcus aureus* in Various Food Samples. Biocatal. Agric. Biotechnol..

[36] Carrascosa C., Raheem D., Ramos F., Saraiva A., Raposo A. (2021). Microbial Biofilms in the Food Industry—A Comprehensive Review. Int. J. Environ. Res. Public Health.

[37] Singh R., Ray P., Das A., Sharma M. (2009). Role of Persisters and Small-Colony Variants in Antibiotic Resistance of Planktonic and Biofilm-Associated *Staphylococcus aureus*: An in Vitro Study. J. Med. Microbiol..

[38] Curtis T.D., Gram L., Knudsen G.M. (2016). The Small Colony Variant of *Listeria monocytogenes* Is More Tolerant to Antibiotics and Has Altered Survival in RAW 264.7 Murine Macrophages. Front. Microbiol..

[39] Frenzel E., Kranzler M., Stark T.D., Hofmann T., Ehling-Schulz M. (2015). The Endospore-Forming Pathogen *Bacillus cereus* Exploits a Small Colony Variant-Based Diversification Strategy in Response to Aminoglycoside Exposure. mBio.

[40] Besse A., Groleau M.-C., Déziel E. (2023). Emergence of Small Colony Variants Is an Adaptive Strategy Used by *Pseudomonas aeruginosa* to Mitigate the Effects of Redox Imbalance. mSphere.

[41] Glover W.A., Yang Y., Zhang Y. (2009). Insights into the Molecular Basis of L-Form Formation and Survival in *Escherichia coli*. PLoS ONE.

[42] Liu S., Brul S., Zaat S.A.J. (2020). Bacterial Persister-Cells and Spores in the Food Chain: Their Potential Inactivation by Antimicrobial Peptides (AMPs). Int. J. Mol. Sci..

[43] Fu Y., Liang L., Deng S., Wu Y., Yuan Y., Gao M. (2020). Novel Spore Lytic Enzyme from a Bacillus Phage Leading to Spore Killing. Enzym. Microb. Technol..

[44] Fernandes S., Gomes I.B., Sousa S.F., Simões M. (2022). Antimicrobial Susceptibility of Persister Biofilm Cells of *Bacillus cereus* and *Pseudomonas fluorescens*. Microorganisms.

[45] Navaneethan Y., Effarizah M.E. (2023). Post-Cooking Growth and Survival of *Bacillus cereus* Spores in Rice and Their Enzymatic Activities Leading to Food Spoilage Potential. Foods.

[46] Lyashchuk Y.O., Novak A.I., Kostrova Y.B., Shibarshina O.Y., Evdokimova O.V., Kanina I.V. (2021). The Study of Persistence of Microorganisms and Parasites in Food Products. IOP Conf. Ser. Earth Environ. Sci..

[47] Osek J., Lachtara B., Wieczorek K. (2022). *Listeria monocytogenes*—How This Pathogen Survives in Food-Production Environments?. Front. Microbiol..

[48] Hillig N., Hamedy A., Koethe M. (2023). *Listeria monocytogenes* Detection on Food Contact Surfaces: Suitability of Different Swab Materials. J. Consum. Prot. Food Saf..

[49] Harter E., Wagner E.M., Zaiser A., Halecker S., Wagner M., Rychli K. (2017). Stress Survival Islet 2, Predominantly Present in *Listeria monocytogenes* Strains of Sequence Type 121, Is Involved in the Alkaline and Oxidative Stress Responses. Appl. Environ. Microbiol..

[50] Narimisa N., Kalani B.S., Mohammadzadeh R., Jazi F.M. (2021). Combination of Antibiotics-Nisin Reduces the Formation of Persister Cell in *Listeria monocytogenes*. Microb. Drug Resist..

[51] Li X., Hospital X.F., Hierro E., Fernández M., Sheng L., Wang L. (2023). Formation of *Listeria monocytogenes* Persister Cells in the Produce-Processing Environment. Int. J. Food Microbiol..

[52] Tuytschaever T., Raes K., Sampers I. (2023). *Listeria monocytogenes* in Food Businesses: From Persistence Strategies to Intervention/Prevention Strategies—A Review. Compr. Rev. Food Sci. Food Saf..

[53] Belias A., Sullivan G., Wiedmann M., Ivanek R. (2022). Factors That Contribute to Persistent Listeria in Food Processing Facilities and Relevant Interventions: A Rapid Review. Food Control.

[54] Miao J., Lin S., Soteyome T., Peters B.M., Li Y., Chen H., Su J., Li L., Li B., Xu Z. (2019). Biofilm Formation of *Staphylococcus aureus* under Food Heat Processing Conditions: First Report on CML Production within Biofilm. Sci. Rep..

[55] Conlon B.P., Rowe S.E., Gandt A.B., Nuxoll A.S., Donegan N.P., Zalis E.A., Clair G., Adkins J.N., Cheung A.L., Lewis K. (2016). Persister Formation in *Staphylococcus aureus* Is Associated with ATP Depletion. Nat. Microbiol..

[56] Morcrette H., Kovacs-Simon A., Tennant R.K., Love J., Wagley S., Yang Z.R., Studholme D.J., Soyer O.S., Champion O.L., Butler C.S. (2020). Campylobacter Jejuni 11168H Exposed to Penicillin Forms Persister Cells and Cells with Altered Redox Protein Activity. Front. Cell Infect. Microbiol..

[57] Ovsepian A., Larsen M.H., Vegge C.S., Ingmer H. (2020). Ciprofloxacin-Induced Persister-Cells in Campylobacter Jejuni. Microbiology.

[58] (2018). Food Safety Management Systems—A Practical Guide.

[59] National Advisory Committee on Microbiological Criteria for Foods HACCP Principles & Application Guidelines. https://www.fda.gov/food/hazard-analysis-critical-control-point-haccp/haccp-principles-application-guidelines.

[60] Khan F., Pham D.T.N., Tabassum N., Oloketuyi S.F., Kim Y.M. (2020). Treatment Strategies Targeting Persister Cell Formation in Bacterial Pathogens. Crit. Rev. Microbiol..

[61] Tran V.N., Dasagrandhi C., Truong V.G., Kim Y.M., Kang H.W. (2018). Antibacterial Activity of *Staphylococcus aureus* Biofilm under Combined Exposure of Glutaraldehyde, near-Infrared Light, and 405-Nm Laser. PLoS ONE.

[62] Manivasagan P., Khan F., Hoang G., Mondal S., Kim H., Hoang Minh Doan V., Kim Y.M., Oh J. (2019). Thiol Chitosan-Wrapped Gold Nanoshells for near-Infrared Laser-Induced Photothermal Destruction of Antibiotic-Resistant Bacteria. Carbohydr. Polym..

[63] Dash K.K., Fayaz U., Dar A.H., Shams R., Manzoor S., Sundarsingh A., Deka P., Khan S.A. (2022). A Comprehensive Review on Heat Treatments and Related Impact on the Quality and Microbial Safety of Milk and Milk-Based Products. Food Chem. Adv..

[64] Rifna E.J., Singh S.K., Chakraborty S., Dwivedi M. (2019). Effect of Thermal and Non-Thermal Techniques for Microbial Safety in Food Powder: Recent Advances. Food Res. Int..

[65] Khalid W., Maggiolino A., Kour J., Arshad M.S., Aslam N., Afzal M.F., Meghwar P., Zafar K.-W., De Palo P., Korma S.A. (2023). Dynamic Alterations in Protein, Sensory, Chemical, and Oxidative Properties Occurring in Meat during Thermal and Non-Thermal Processing Techniques: A Comprehensive Review. Front. Nutr..

[66] Srivastava P.K., Sit N. (2024). A Review on Fruit and Vegetable Processing Using Traditional and Novel Methods. Futur. Postharvest Food.

[67] Catherine M.G.C., Renard J.F.M., Sun D.-W. (2012). Thermal Food Processing New Technologies and Qualities Issues: Thermal Processing of Fruits and Fruit Juices.

[68] Evelyn, Silva F.V.M. (2015). High Pressure Processing of Milk: Modeling the Inactivation of Psychrotrophic *Bacillus cereus* Spores at 38–70 °C. J. Food Eng..

[69] Luu-Thi H., Corthouts J., Passaris I., Grauwet T., Aertsen A., Hendrickx M., Michiels C.W. (2015). Carvacrol Suppresses High Pressure High Temperature Inactivation of *Bacillus cereus* Spores. Int. J. Food Microbiol..

[70] Santos L.M., Oliveira F.A., Ferreira E.H., Rosenthal A. (2017). Application and Possible Benefits of High Hydrostatic Pressure or High-Pressure Homogenization on Beer Processing: A Review. Food Sci. Technol. Int..

[71] Aganovic K., Hertel C., Vogel R.F., Johne R., Schlüter O., Schwarzenbolz U., Jäger H., Holzhauser T., Bergmair J., Roth A. (2021). Aspects of High Hydrostatic Pressure Food Processing: Perspectives on Technology and Food Safety. Compr. Rev. Food Sci. Food Saf..

[72] Huang H.-W., Wu S.-J., Lu J.-K., Shyu Y.-T., Wang C.-Y. (2017). Current Status and Future Trends of High-Pressure Processing in Food Industry. Food Control.

[73] Naveena B., Nagaraju M. (2020). Review on Principles, Effects, Advantages and Disadvantages of High Pressure Processing of Food. Int. J. Chem. Stud..

[74] Niepa T.H.R., Gilbert J.L., Ren D. (2012). Controlling *Pseudomonas aeruginosa* Persister Cells by Weak Electrochemical Currents and Synergistic Effects with Tobramycin. Biomaterials.

[75] Niepa T.H.R., Snepenger L.M., Wang H., Sivan S., Gilbert J.L., Jones M.B., Ren D. (2016). Sensitizing *Pseudomonas aeruginosa* to Antibiotics by Electrochemical Disruption of Membrane Functions. Biomaterials.

[76] (2019). USFDA CFR—Code of Federal Regulations Title 21 the Information on This Page Is Current as of 1 April 2016. https://www.ecfr.gov.

[77] Liu D., Huang Q., Gu W., Zeng X.-A. (2022). A Review of Bacterial Biofilm Control by Physical Strategies. Crit. Rev. Food Sci. Nutr..

[78] Soro A.B., Whyte P., Bolton D.J., Tiwari B.K. (2020). Strategies and Novel Technologies to Control Campylobacter in the Poultry Chain: A Review. Compr. Rev. Food Sci. Food Saf..

[79] Schmidt R.H. (2018). Basic Elements of Equipment Cleaning and Sanitizing in Food Processing and Handling Operations 1.

[80] Bayoumi M.A., Kamal R.M., Abd El Aal S.F., Awad E.I. (2012). Assessment of a Regulatory Sanitization Process in Egyptian Dairy Plants in Regard to the Adherence of Some Food-Borne Pathogens and Their Biofilms. Int. J. Food Microbiol..

[81] Atasoy M., Álvarez Ordóñez A., Cenian A., Djukić-Vuković A., Lund P.A., Ozogul F., Trček J., Ziv C., De Biase D. (2024). Exploitation of Microbial Activities at Low PH to Enhance Planetary Health. FEMS Microbiol. Rev..

[82] Wood J.M. (2015). Bacterial Responses to Osmotic Challenges. J. Gen. Physiol..

[83] Mari A., Parisouli D.N., Krokida M. (2024). Exploring Osmotic Dehydration for Food Preservation: Methods, Modelling, and Modern Applications. Foods.

[84] Xiong X., Kong J., Qi D., Xiong X., Liu Y., Cui X. (2022). Presence, Formation, and Elimination of Foodborne Pathogen Persisters. JSFA Rep..

[85] Karki P., Mohiuddin S.G., Kavousi P., Orman M.A. (2020). Investigating the Effects of Osmolytes and Environmental PH on Bacterial Persisters. Antimicrob. Agents Chemother..

[86] Aryal M., Muriana P.M. (2019). Efficacy of Commercial Sanitizers Used in Food Processing Facilities for Inactivation of *Listeria monocytogenes*, *E. coli* O157:H7, and *Salmonella* Biofilms. Foods.

[87] Duze S.T., Marimani M., Patel M. (2021). Tolerance of *Listeria monocytogenes* to Biocides Used in Food Processing Environments. Food Microbiol..

[88] Lioy V.S., Rey O., Balsa D., Pellicer T., Alonso J.C. (2010). A Toxin–Antitoxin Module as a Target for Antimicrobial Development. Plasmid.

[89] Park S.J., Son W.S., Lee B.-J. (2013). Structural Overview of Toxin–Antitoxin Systems in Infectious Bacteria: A Target for Developing Antimicrobial Agents. Biochim. Biophys. Acta-Proteins Proteom..

[90] Barraud N., Buson A., Jarolimek W., Rice S.A. (2013). Mannitol Enhances Antibiotic Sensitivity of Persister Bacteria in *Pseudomonas aeruginosa* Biofilms. PLoS ONE.

[91] Leszczynska D., Matuszewska E., Kuczynska-Wisnik D., Furmanek-Blaszk B., Laskowska E. (2013). The Formation of Persister Cells in Stationary-Phase Cultures of *Escherichia coli* Is Associated with the Aggregation of Endogenous Proteins. PLoS ONE.

[92] Mizzi L., Maniscalco D., Gaspari S., Chatzitzika C., Gatt R., Valdramidis V.P. (2020). Assessing the Individual Microbial Inhibitory Capacity of Different Sugars against Pathogens Commonly Found in Food Systems. Lett. Appl. Microbiol..

[93] Msomi N.Z., Erukainure O.L., Islam M.S. (2021). Suitability of Sugar Alcohols as Antidiabetic Supplements: A Review. J. Food Drug Anal..

[94] Medina-Rodríguez A.C., Ávila-Sierra A., Ariza J.J., Guillamón E., Baños-Arjona A., Vicaria J.M., Jurado E. (2020). Clean-in-Place Disinfection of Dual-Species Biofilm (*Listeria* and *Pseudomonas*) by a Green Antibacterial Product Made from Citrus Extract. Food Control.

[95] Cacciatore F.A., Brandelli A., Malheiros P.d.S. (2021). Combining Natural Antimicrobials and Nanotechnology for Disinfecting Food Surfaces and Control Microbial Biofilm Formation. Crit. Rev. Food Sci. Nutr..

[96] Stojanović-Radić Z., Pejčić M., Joković N., Jokanović M., Ivić M., Šojić B., Škaljac S., Stojanović P., Mihajilov-Krstev T. (2018). Inhibition of *Salmonella* Enteritidis Growth and Storage Stability in Chicken Meat Treated with Basil and Rosemary Essential Oils Alone or in Combination. Food Control.

[97] Jokanović M., Ivić M., Škaljac S., Tomović V., Pavlić B., Šojić B., Zeković Z., Peulić T., Ikonić P. (2020). Essential Oil and Supercritical Extracts of Winter Savory (*Satureja montana* L.) as Antioxidants in Precooked Pork Chops during Chilled Storage. LWT.

[98] Tomović V., Šojić B., Savanović J., Kocić-Tanackov S., Pavlić B., Jokanović M., Đorđević V., Parunović N., Martinović A., Vujadinović D. (2022). Caraway (*Carum carvi* L.) Essential Oil Improves Quality of Dry-fermented Sausages Produced with Different Levels of Sodium Nitrite. J. Food Process Preserv..

[99] Muruzović M., Mladenović K.G., Stefanović O.D., Vasić S.M., Čomić L.R. (2016). Extracts of *Agrimonia eupatoria* L. as Sources of Biologically Active Compounds and Evaluation of Their Antioxidant, Antimicrobial, and Antibiofilm Activities. J. Food Drug Anal..

[100] Lu M., Wang S., Wang T., Hu S., Bhayana B., Ishii M., Kong Y., Cai Y., Dai T., Cui W. (2021). Bacteria-Specific Phototoxic Reactions Triggered by Blue Light and Phytochemical Carvacrol. Sci. Transl. Med..

[101] Laranjo M., Elias M., Fraqueza M.J. (2017). The Use of Starter Cultures in Traditional Meat Products. J. Food Qual..

[102] Grujović M.Ž., Mladenović K.G., Semedo-Lemsaddek T., Laranjo M., Stefanović O.D., Kocić-Tanackov S.D. (2022). Advantages and Disadvantages of Non-starter Lactic Acid Bacteria from Traditional Fermented Foods: Potential Use as Starters or Probiotics. Compr. Rev. Food Sci. Food Saf..

[103] Le C.-F., Fang C.-M., Sekaran S.D. (2017). Intracellular Targeting Mechanisms by Antimicrobial Peptides. Antimicrob. Agents Chemother..

[104] Henderson L.O., Erazo Flores B.J., Skeens J., Kent D., Murphy S.I., Wiedmann M., Guariglia-Oropeza V. (2020). Nevertheless, She Resisted—Role of the Environment on *Listeria monocytogenes* Sensitivity to Nisin Treatment in a Laboratory Cheese Model. Front. Microbiol..

[105] Gut I.M., Blanke S.R., van der Donk W.A. (2011). Mechanism of Inhibition of *Bacillus anthracis* Spore Outgrowth by the Lantibiotic Nisin. ACS Chem. Biol..

[106] Balandin S.V., Sheremeteva E.V., Ovchinnikova T.V. (2019). Pediocin-Like Antimicrobial Peptides of Bacteria. Biochemistry.

[107] Rishi P., Bhagat N.R., Thakur R., Pathania P. (2018). Tackling *Salmonella* Persister Cells by Antibiotic–Nisin Combination via Mannitol. Indian J. Microbiol..

[108] Lin D.M., Koskella B., Lin H.C. (2017). Phage Therapy: An Alternative to Antibiotics in the Age of Multi-Drug Resistance. World J. Gastrointest. Pharmacol. Ther..

[109] Caflisch K.M., Patel R. (2019). Implications of Bacteriophage- and Bacteriophage Component-Based Therapies for the Clinical Microbiology Laboratory. J. Clin. Microbiol..

[110] Roach D.R., Donovan D.M. (2015). Antimicrobial Bacteriophage-Derived Proteins and Therapeutic Applications. Bacteriophage.

[111] Schuch R., Khan B.K., Raz A., Rotolo J.A., Wittekind M. (2017). Bacteriophage Lysin CF-301, a Potent Antistaphylococcal Biofilm Agent. Antimicrob. Agents Chemother..

[112] Singh A., Padmesh S., Dwivedi M., Kostova I. (2022). How Good Are Bacteriophages as an Alternative Therapy to Mitigate Biofilms of Nosocomial Infections. Infect. Drug Resist..

[113] Fister S., Robben C., Witte A.K., Schoder D., Wagner M., Rossmanith P. (2016). Influence of Environmental Factors on Phage-Bacteria Interaction and on the Efficacy and Infectivity of Phage P100. Front. Microbiol..

[114] Iacumin L., Manzano M., Comi G. (2016). Phage Inactivation of *Listeria monocytogenes* on San Daniele Dry-Cured Ham and Elimination of Biofilms from Equipment and Working Environments. Microorganisms.

[115] Gutiérrez D., Ruas-Madiedo P., Martínez B., Rodríguez A., García P. (2014). Effective Removal of Staphylococcal Biofilms by the Endolysin LysH5. PLoS ONE.

[116] Briers Y., Walmagh M., Grymonprez B., Biebl M., Pirnay J.P., Defraine V., Michiels J., Cenens W., Aertsen A., Miller S. (2014). Art-175 Is a Highly Efficient Antibacterial against Multidrug-Resistant Strains and Persisters of *Pseudomonas aeruginosa*. Antimicrob. Agents Chemother..

[117] Defraine V., Schuermans J., Grymonprez B., Govers S.K., Aertsen A., Fauvart M., Michiels J., Lavigne R., Briers Y. (2016). Efficacy of Artilysin Art-175 against Resistant and Persistent *Acinetobacter baumannii*. Antimicrob. Agents Chemother..

[118] Chung E.S., Ko K.S. (2019). Eradication of Persister Cells of *Acinetobacter baumannii* through Combination of Colistin and Amikacin Antibiotics. J. Antimicrob. Chemother..

[119] Nahar S., Mizan M.F.R., Ha A.J., Ha S. (2018). Do Advances and Future Prospects of Enzyme-Based Biofilm Prevention Approaches in the Food Industry. Compr. Rev. Food Sci. Food Saf..

[120] Meireles A., Borges A., Giaouris E., Simões M. (2016). The Current Knowledge on the Application of Anti-Biofilm Enzymes in the Food Industry. Food Res. Int..

[121] Sikdar R., Elias M. (2020). Quorum Quenching Enzymes and Their Effects on Virulence, Biofilm, and Microbiomes: A Review of Recent Advances. Expert Rev. Anti. Infect. Ther..

[122] Salman M.K., Abuqwider J., Mauriello G. (2023). Anti-Quorum Sensing Activity of Probiotics: The Mechanism and Role in Food and Gut Health. Microorganisms.

[123] Zawawi N.A.F., Hazmi N.A.M., How M.S., Kantono K., Silva F.V.M., Sulaiman A. (2022). Thermal, High Pressure, and Ultrasound Inactivation of Various Fruit Cultivars’ Polyphenol Oxidase: Kinetic Inactivation Models and Estimation of Treatment Energy Requirement. Appl. Sci..

[124] Dai Y., Chen Y., Lin X., Zhang S. (2024). Recent Applications and Prospects of Enzymes in Quality and Safety Control of Fermented Foods. Foods.

[125] Abril B., Bou R., García-Pérez J.V., Benedito J. (2023). Role of Enzymatic Reactions in Meat Processing and Use of Emerging Technologies for Process Intensification. Foods.

[126] Lopes N.A., Pinilla C.M.B., Brandelli A. (2017). Pectin and Polygalacturonic Acid-Coated Liposomes as Novel Delivery System for Nisin: Preparation, Characterization and Release Behavior. Food Hydrocoll..

[127] Srividya N., Ghoora M.D., Padmanabh P.R. (2017). Antimicrobial Nanotechnology: Research Implications and Prospects in Food Safety. Food Preservation.

[128] Jildeh Z.B., Wagner P.H., Schöning M.J. (2021). Sterilization of Objects, Products, and Packaging Surfaces and Their Characterization in Different Fields of Industry: The Status in 2020. Phys. Status Solidi Appl. Mater. Sci..

[129] Chiozzi V., Agriopoulou S., Varzakas T. (2022). Advances, Applications, and Comparison of Thermal (Pasteurization, Sterilization, and Aseptic Packaging) against Non-Thermal (Ultrasounds, UV Radiation, Ozonation, High Hydrostatic Pressure) Technologies in Food Processing. Appl. Sci..

[130] Abana C.M., Brannon J.R., Ebbott R.A., Dunigan T.L., Guckes K.R., Fuseini H., Powers J., Rogers B.R., Hadjifrangiskou M. (2017). Characterization of Blue Light Irradiation Effects on Pathogenic and Nonpathogenic *Escherichia coli*. Microbiologyopen.

[131] Marques A.P., Santos C., Sério J., Crespo M.T.B., Pereira V.J. (2024). Enhancing Food Safety: Employing Ultraviolet-C Light Emitting Diodes for Water, Leaf, and Surface Disinfection. Innov. Food Sci. Emerg. Technol..

[132] Malka S.K., Park M.-H. (2022). Fresh Produce Safety and Quality: Chlorine Dioxide’s Role. Front. Plant Sci..

[133] Song P., Zhang X., Wang S., Xu W., Wang F., Fu R., Wei F. (2023). Microbial Proteases and Their Applications. Front. Microbiol..

[134] Zoellner C., Aguayo-Acosta A., Siddiqui M.W., Dávila-Aviña J.E., Siddiqui M.W. (2018). Peracetic Acid in Disinfection of Fruits and Vegetables. Postharvest Disinfection of Fruits and Vegetables.

[135] Terpou A., Papadaki A., Lappa I., Kachrimanidou V., Bosnea L., Kopsahelis N. (2019). Probiotics in Food Systems: Significance and Emerging Strategies Towards Improved Viability and Delivery of Enhanced Beneficial Value. Nutrients.

[136] Liu Y., Sameen D.E., Ahmed S., Dai J., Qin W. (2021). Antimicrobial Peptides and Their Application in Food Packaging. Trends Food Sci. Technol..

[137] Yuan L., Sadiq F.A., Wang N., Yang Z., He G. (2021). Recent Advances in Understanding the Control of Disinfectant-Resistant Biofilms by Hurdle Technology in the Food Industry. Crit. Rev. Food Sci. Nutr..

[138] Tkhilaishvili T., Lombardi L., Klatt A.B., Trampuz A., Di Luca M. (2018). Bacteriophage Sb-1 Enhances Antibiotic Activity against Biofilm, Degrades Exopolysaccharide Matrix and Targets Persisters of *Staphylococcus aureus*. Int. J. Antimicrob. Agents.

[139] Lu T.K., Collins J.J. (2009). Engineered Bacteriophage Targeting Gene Networks as Adjuvants for Antibiotic Therapy. Proc. Natl. Acad. Sci. USA.

[140] Bahrami A., Delshadi R., Jafari S.M., Williams L. (2019). Nanoencapsulated Nisin: An Engineered Natural Antimicrobial System for the Food Industry. Trends Food Sci. Technol..

